# Phylogeny and species delimitation in Silene sect. Arenosae (Caryophyllaceae): a new section

**DOI:** 10.3897/phytokeys.159.51500

**Published:** 2020-09-04

**Authors:** Frida Eggens, Farzaneh Jafari, Mikael Thollesson, Simon Crameri, Shahin Zarre, Bengt Oxelman

**Affiliations:** 1 Department of Systematic Botany, Evolutionary Biology Centre, Uppsala University, Norbyvägen 18D, SE-752 36 Uppsala, Sweden; 2 Centre of Excellence in Phylogeny of Living Organisms, and Department of Plant Science, School of Biology, College of Science, University of Tehran, 14155–6455, Tehran, Iran; 3 Department of Biological and Environmental Sciences, University of Gothenburg, 40530 Gothenburg, Sweden; 4 Department of Molecular Evolution, Evolutionary Biology Centre, Uppsala University, Norbyvägen 18C, SE-752 36 Uppsala, Sweden; 5 Institute of Integrative Biology, ETH Zurich, Zurich, Switzerland; 6 Gothenburg Global Biodiversity Centre, University of Gothenburg, P.O. Box 461, 40530 Göteborg, Sweden

**Keywords:** Caryophyllaceae, integrative taxonomy, phylogenetics, Plant taxonomy, *
Silene
*, systematics, taxonomic description models

## Abstract

A putatively monophyletic group of annual *Silene* species is revised taxonomically and described as the new section S. sect. Arenosae. The species of this section were previously treated as a part of a widely circumscribed and polyphyletic S. sect. Rigidulae. Silene sect. Arenosae as circumscribed here consists of nine species. Members of the section show a predominantly E Mediterranean to SW Asian distribution pattern from Turkey southward to Egypt and eastward to Iran and Pakistan, although most of the species have a limited distribution range. The species of S. sect. Arenosae are characterized by narrowly lanceolate calyx teeth, which are often highly polymorphic, and lanceolate to oblanceolate (non-spathulate) basal leaves. The provided taxonomic revision is based on morphological characters and supported by phylogenetic analyses of two nuclear loci (nrITS and an intron of the *RPB2* gene) and one chloroplast locus (the intron of the *rps16* gene). The species descriptions are formalized using a novel implementation of the Prometheus Description Model.

## Introduction

*Silene* L. is a large genus of the family Caryophyllaceae, with around 870 currently (Jafari et al. 2020) recognized species that are mainly distributed in the northern hemisphere, South Africa and South America, in temperate to arctic regions and a wide range of habitats (Manning and Goldblatt 2012, Frajman et al. 2018, Jafari et al. 2020). Chowdhuri (1957) delimited 44 sections and his taxonomy has been applied by authors of local floras in the Mediterranean region and SW Asia, including Palestine (Zohary 1966), Turkey (Coode and Cullen 1967), the Iranian Highlands (Melzheimer 1988), the Flora Europaea (Chater et al. 1993), and Iraq (Townsend et al. 2016). There have been several regionally focused studies (e.g., Greuter 1995, Oxelman and Greuter 1997) that amended the taxonomy of Chowdhuri (1957), and a number of molecular studies (e.g., Oxelman and Lidén 1995, Desfeux and Lejeune 1996, Oxelman et al. 1997, Popp and Oxelman 2004, Eggens 2006, Eggens et al. 2007, Popp and Oxelman 2007, Petri and Oxelman 2011, Rautenberg et al. 2012, Aydin et al. 2014a, Naciri et al. 2017) that revealed the artificial nature of many sections as defined by Chowdhuri (1957). Jafari et al. (2020) outlined a new, revised system taking the phylogenetic information into account.

Silene sect. Rigidulae (Boiss.) Schischk. as traditionally circumscribed is superficially coherent morphologically (Eggens 2006). Boissier (1867) first introduced *Rigidulae* as an unranked group (indicated as ‘§’) with 13 species. In a monograph, Rohrbach (1868) accepted this group as a series and classified 20 species in S. ser. Rigidulae (Boiss.) Rohrb. Schischkin (1936) was the first to apply the rank of section for these species. Chowdhuri (1957) subsequently assigned 14 species from the Mediterranean area and SW Asia, Russia and India, to S. sect. Rigidulae, following a similar circumscription to that of Boissier (1867). Greuter (1995) included four Greek species in S. sect. Rigidulae and made a correction on the section’s typification. Molecular phylogenetic data from three putatively unlinked genes revealed that the widely circumscribed S. sect. Rigidulae sensu Chowdhuri (1957) is not monophyletic, but rather consists of at least six independent lineages, each with a fairly good correlation with geography (Eggens 2006). One of the clades recognized in Eggens (2006) comprises taxa found in SW Asia including Turkey, Armenia, Egypt and the Arabian Peninsula, and extending eastwards to Pakistan. This clade, referred to as the “Middle East Clade” in Eggens (2006), is a strongly supported monophyletic group with associated morphological characters (often densely ciliate and lanceolate calyx teeth, and often oblanceolate rather than spathulate basal leaves) that distinguish them from other taxa earlier assigned to S. sect. Rigidulae sensu Chowdhuri (1957). In the present study we refer to this clade as the “SW Asian Clade”.

In this paper, we present morphological, phylogenetic and geographical data on the “SW Asian Clade” that accumulated since Eggens (2006). We integrate all the available evidence and formally describe the “SW Asian Clade” as Silene sect. Arenosae Eggens, F. Jafari & Oxelman, sect. nov., which we consider as one out of several lineages of a polyphyletic S. sect. Rigidulae sensu lato. We provide an identification key and taxonomic revision of all species of the new section, and also place it in a wider phylogenetic context.

## Materials and methods

### Taxon sampling and molecular data

The specimens from the following herbaria: B, BM, BSB, C, E, G, GB, K, LD, LE, S, TUB, UPS, W, WAG and WU (abbreviations according to Thiers 2019+) were used for morphological studies and DNA extraction.

We generated a species tree phylogeny based on three putatively unlinked loci and used the species tree as a framework for our taxonomic revision. The advantage of using monophyletic groups as a starting point for taxonomic revisions in complex genera such as *Silene* is that parallelism and character reversals can be better understood in the search for diagnostic morphological characters. The species tree is based on sequences from three regions: the nuclear ribosomal internal transcribed spacers (nrITS, with the intervening 5.8S gene), the second last intron of the nuclear *RPB2* gene (Popp and Oxelman 2004), and the intron of the chloroplast gene *rps16* (Oxelman et al. 1997).

The phylogenetic study is based on 84 sequences from 55 species representing two subgenera of *Silene*, *Behenantha* (Otth) Torr. & A.Gray and *Silene* with 39 sequences of *RPB2* region being generated for the purpose of this paper. Material used for the phylogenetic analyses are presented in Suppl. material 1. The procedures for extraction of total genomic DNA, amplification of the DNA regions by the polymerase chain reaction, sequencing reactions and their visualization were described in Eggens et al. (2007). All sequences were edited using Sequencher 3.1.1 (Gene Codes Corporation) and aligned manually with Aliview (Larsson 2014) following criteria presented in Eggens et al. (2007).

### Phylogenetic analyses

Maximum Parsimony (MP) analyses of individual multiple alignments were performed with PAUP* v.4.0a162 (Swofford 2018). Heuristic searches employed 100 random addition sequences, TBR (tree-bisection-reconnection) branch-swapping algorithm. Maximum parsimony bootstrap (MPB) percentages were calculated with the parameters: hsearch addseq = random, nchuck = 2, chuckscore = 600, nreps = 1, bootstrap nreps = 1000 (summarized in a 50% majority-rule consensus tree). PAUP* 4.0a162 (Swofford 2018) was used to select the best-fitted model of nucleotide substitution based on the Akaike information criteria corrected (AICc), and the General Time Reversible model with Gamma shaped rate variation (GTR+G) model was selected for all three regions. Maximum likelihood (ML) analyses were conducted in RAxML HPC v.8.2.10 (Stamatakis 2014) using GTRGAMMA model with 1000 pseudo-replicates to evaluate bootstrap support for each node. Bayesian gene tree inference was performed using MrBayes v.3.2.6 (Ronquist et al. 2012) with 20 million generations for each of the three datasets. Four Metropolis-coupled chains were run with trees and parameter values saved every 1000^th^ generations in two parallel runs. The first 25% of total trees were discarded as burn-in.

Species tree analyses were performed with STACEY (Species Tree And Classification Estimation, Yarely) v.1.2.5 (Jones 2016) as implemented in BEAST v.2.5.1 (Bouckaert et al. 2014, 2019). All specimens where we had access to sequences from at least two of the regions were included in the species tree analysis. An input file was created with BEAUTi v.2.5.1 in which substitution models, clock models and gene trees for all loci were unlinked. The General Time Reversible (GTR) substitution model with rate variation following a gamma distribution with four rate categories, a relaxed lognormal clock and fixed average clock rate for one arbitrary locus set to 1 were chosen. The ploidy level was set to 1 for ITS and *rps16* partitions, and 2 for the nuclear *RPB2* locus. The prior growth rate was set to a lognormal distribution with mean 4.6 and standard deviation 2. The popPriorScale was set to a lognormal with mean –7 and standard deviation 2. The prior for ucldMean was set to a log normal distribution with mean 0 and standard deviation 1, otherwise the default priors were applied. The CollapseHeight, which is an approximation of zero node height in the species tree (see Jones et al. 2015) was set to 1E-4. The input ﬁle was run for 250 million iterations by logging every 25000^th^ iterations, with two replicates. Convergence and effective sample size (ESS) values were considered sufficient when each parameter was higher than 200 as verified in Tracer v.1.7 (Rambaut et al. 2018). LogCombiner v.2.5.1 was used to discard the 1000 first trees of each of the two separate runs and then combine the rest of the trees as an estimate of the posterior. Finally, trees were summarized in TreeAnnoatator v.2.5.1. All phylogenetic analyses were carried out on the CIPRES science gateway (Miller et al. 2010).

A similarity matrix representing posterior frequencies of clusters of individuals was produced from the second replicate set of species trees generated with STACEY, using the program SpeciesDelimationAnalyser v.1.2.5 (speciesDA.jar, http://www.indriid.com/software.html) with 10% burn-in and CollapseHeight of 1E-4. The CollapseHeight is an approximation of zero node height (Jones et al. 2015) and individuals clustering together below this height can therefore be considered as belonging to the same ideal population according to the multispecies coalescent model. The estimated similarity matrix was then visualized using the R script plot.simmatrix.R (https://github.com/scrameri/smtools/tree/master/SpeciesDelimitation), which plots a heatmap of the similarity matrix after automatic sorting of rows and columns according to the summary species tree topology.

### Plant descriptions

The species descriptions in this paper are extracted from a database and application (X303) developed based on “Prometheus Description Model” (Pullan et al. 2005) which is a system for handling descriptive data in a digital form. The idea behind this model is to present and store taxonomic information in a way that makes it comparable and exchangeable between different projects. This makes it different from other digitalized description systems, such as DELTA (Dallwitz 1980).

A description in the Prometheus model is built up by descriptive elements (DE) that have three parts – a structure, a property and one or more scores (states for a qualitative property, values for a quantitative property). Additionally, a DE can have modifiers such as frequency (e.g., ‘usually’, ‘sometimes’), relative (e.g., ‘less-than’, ‘equal-to’), spatial (e.g., ‘above’, ‘below’), or temporal (e.g., ‘after’, ‘during’) modifiers. An important component in the Prometheus Model, to make different descriptions exchangeable, is the use of an ontology, i.e. a defined terminology, specifying the different structure and property designations that are allowed in a description. This is applied in two steps: the base ontology, and a description template (pro-forma ontology), which is a derived version of the ontology used for a specific context. For the purpose of this study we started with the published Prometheus basal angiosperm ontology (http://www.dcs.napier.ac.uk/~prometheus/prometheus_2/Resources/Ontology.xml). We found, however, that we needed to both extend the vocabulary, and to make a conceptual extension to the models to enable us to describe the *Silene* taxa adequately. After extracting the preliminary descriptions, we modified them manually for each species, and also provided a general description for S. sect. Arenosae (see “Discussion” under description of the section) that includes all constant features among the species assigned to this section. Using this method, we avoided redundancy.

Some terms missing from the ontology were such structures that are more taxon specific, e.g. ‘anthophore’, used in the sense proposed by Greuter (1995), i.e. a structure that separates the attachment of calyx and corolla. Other (sub-) structures could be described using the available ontology, but only very awkwardly, and we considered it justified to include them as well (e.g., the flower structures ‘limb’ and ‘claw’, the former being the upper part of the petals and the latter the lower part; see also Lawrence 1951, for definition). Some states (e.g., ‘unicellular’ and ‘multicellular’) were also added, although some could have been introduced as structures (e.g., ‘cell’) and used with existing properties.

A more conceptually interesting issue, where we have extended the Prometheus model, is the need to single out a specific structure (e.g., the ‘uppermost’) from a collection of such structures (e.g., ‘internodes’). Pullan et al. (2005) briefly discussed this issue (by using a state of a property to identify a specific structure in a DE), but in our data we found the problem to be more general. Our solution is essentially to use properties and modifiers available in the ontology, but placing them in a specific context, the Specifier Element. The specifier element is a part of the description template associated with a specific instance of the ontology (structure) in question. An example for this case can be represented by the first flower. In a dichasium, there is always a first flower developing before the other flowers. Later flowers and inflorescence branches appear adjacent to the bracts of the first flower. The pedicel of the first flower (in some literature called the alar flower) is longer than the pedicels of later flowers, and as the pedicels continue to grow as long as the plant is alive, “length of pedicel of the first flower (or fruit)” is given as opposed to “length of pedicel” which could apply to any pedicel length.

Links to the descriptions, as well as details on specimens, can be found at the *Sileneae* website available at http://www.sileneae.info (Oxelman et al. 2013). The database itself is stored at http://www.sileneae.info/x303/ and can be viewed by logging in with “guest” as both username and password.

Information on localities was obtained from herbarium labels. When coordinates were not noted on the labels, coordinates were assigned to the locations using the GPS Coordinates network (https://www.gps-coordinates.net), GeoNames (https://www.geonames.org), or FallingRain (http://www.fallingrain.com) servers from information on localities (region, nearby town, etc.) on the labels. Coordinates have been assigned to a representative subset of the material studied, in attempt to provide the geographical distribution maps of the taxa studied.

## Results

The results of our morphological studies are performed in the form of descriptions of the section, species and subspecies under “Discussion”. The phylogenetic results, including alignment characteristics and tree topologies, are presented here.

Some features of the sequence alignments and matrices as well as statistics of the resulting phylogenetic trees are summarized in Table 1.

**Table 1.** Characteristics of the matrices and the resulting trees. (MPT = Most Parsimonious Trees, CI = Consistency Index excluding uninformative characters; RI = Retention Index).

	**Terminals**	**Positions**	**No of MPT trees found**	**Tree length**	**CI**	**RI**
ITS	76	737	115	561	0.4902	0.7925
rps16	71	1053	375	408	0.7598	0.8818
RPB2	76	1385	320	608	0.6617	0.8533

**Figure 1.**
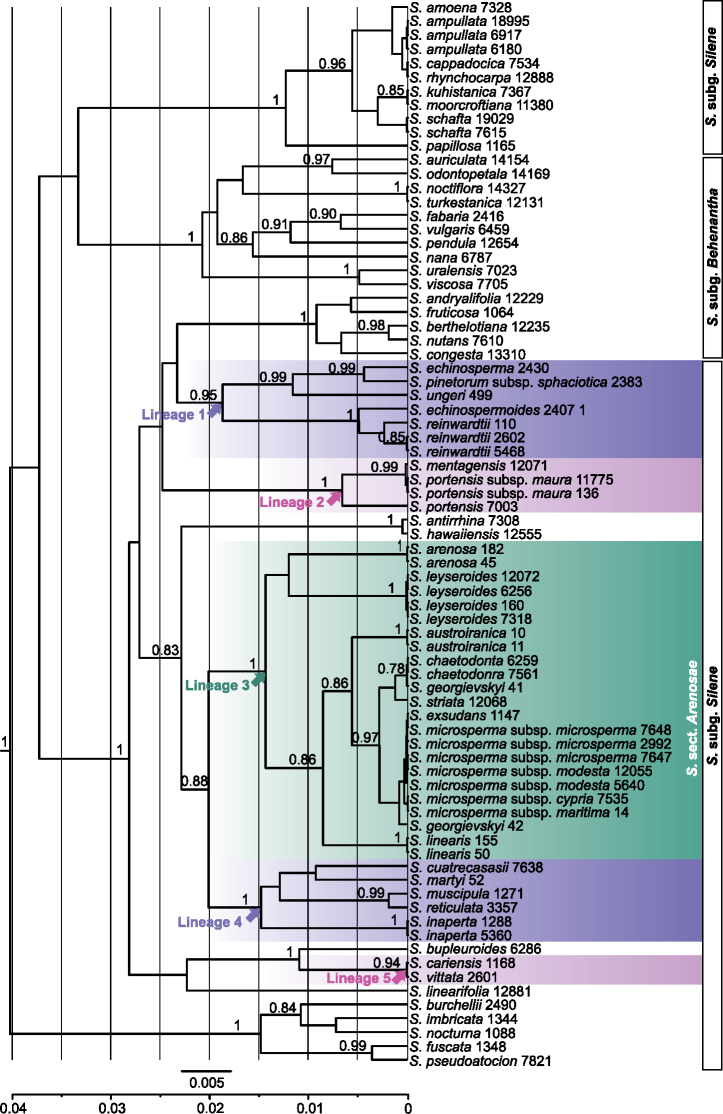
Species tree from two STACEY runs and three unlinked regions (ITS, *RPB2*, *rps16*). Posterior probabilities >0.75 are shown above branches. The number following the taxonomic name is the specimen ID (Suppl. material 1). Scale bar reﬂects the number of substitutions per site.

Silene sect. Arenosae was recovered as monophyletic in the species tree (PP = 1.00, Fig. 1). A clade including some members of S. sect. Rigidulae sensu Chowdhuri (1957), circumscribed as S. sect. Muscipula (Tzvelev) Oxelman, F.Jafari & Gholipour (Jafari et al. 2020), is sister to S. sect. Arenosae in the species tree (PP = 0.88, Fig. 1). *Silene arenosa* K.Koch and *S. leyseroides* Boiss. are poorly resolved at the base of the section in the species tree (Fig. 1). *Silene linearis* Decne. and *S. austroiranica* Rech.f., Aellen & Esfand. form successive sisters with respect to the rest of S. sect. Arenosae (PP = 0.86 and PP = 0.86, Fig. 1).

**Figure 2.**
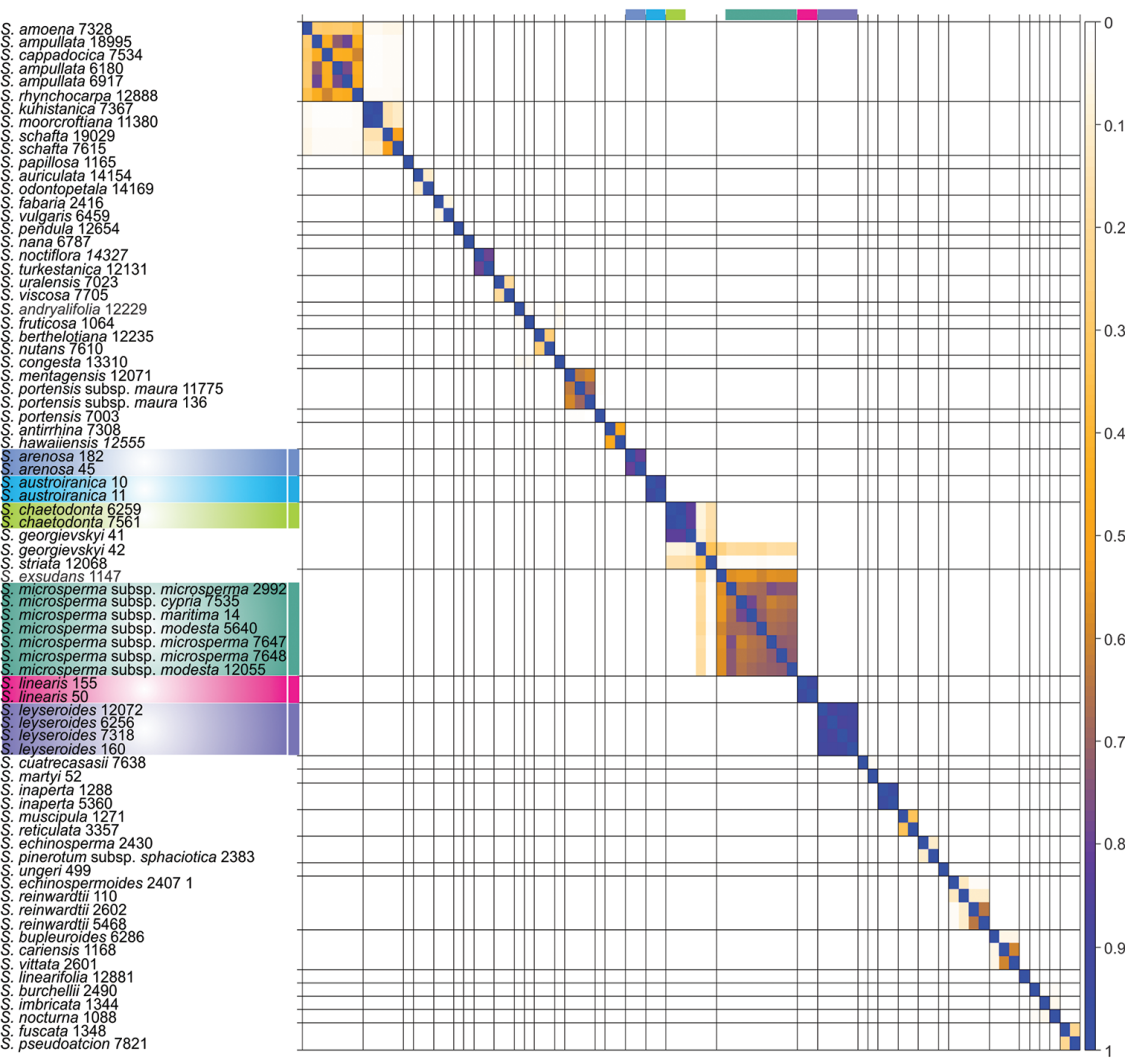
Similarity matrix calculated using SpeciesDelimationAnalyser v.1.2.5 (speciesDA.jar, http://www.indriid.com/software.html).

**Figure 3.**
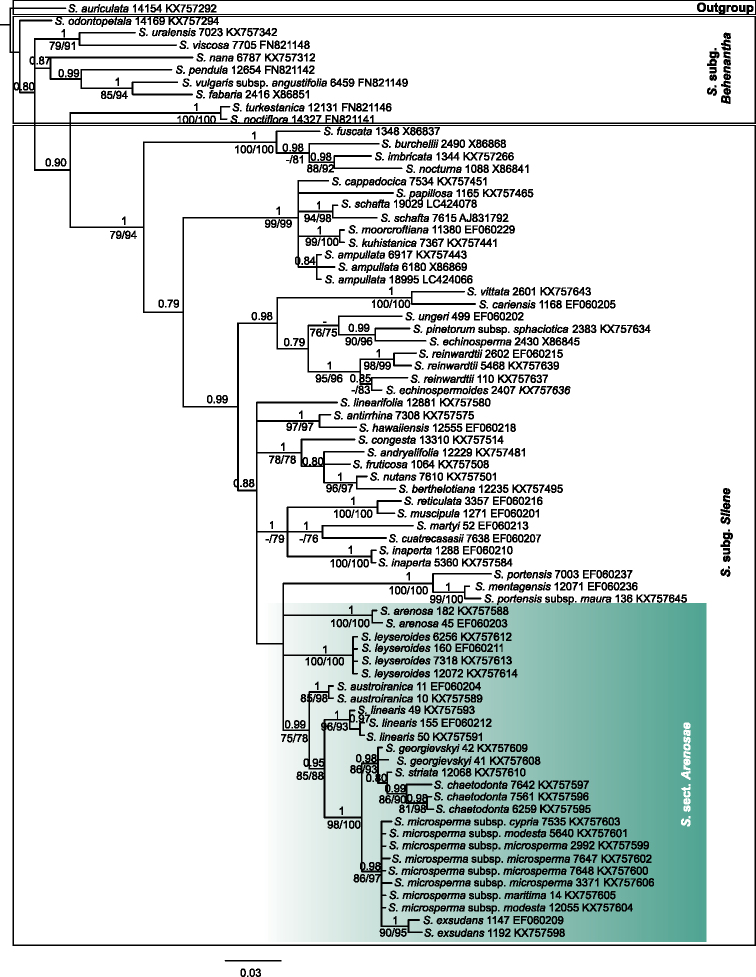
Phylogenetic tree resulting from Bayesian analysis of the ITS sequences including 76 taxa. The trees were summarized in a 50% majority-rule consensus tree with the posterior probabilities (PP) indicated above branches. Bootstrap support values (>75%) based on MP and ML are noted below branches, respectively. The numbers following the taxonomic name indicate the specimen ID and Genbank numbers (Suppl. material 1), respectively.

**Figure 4.**
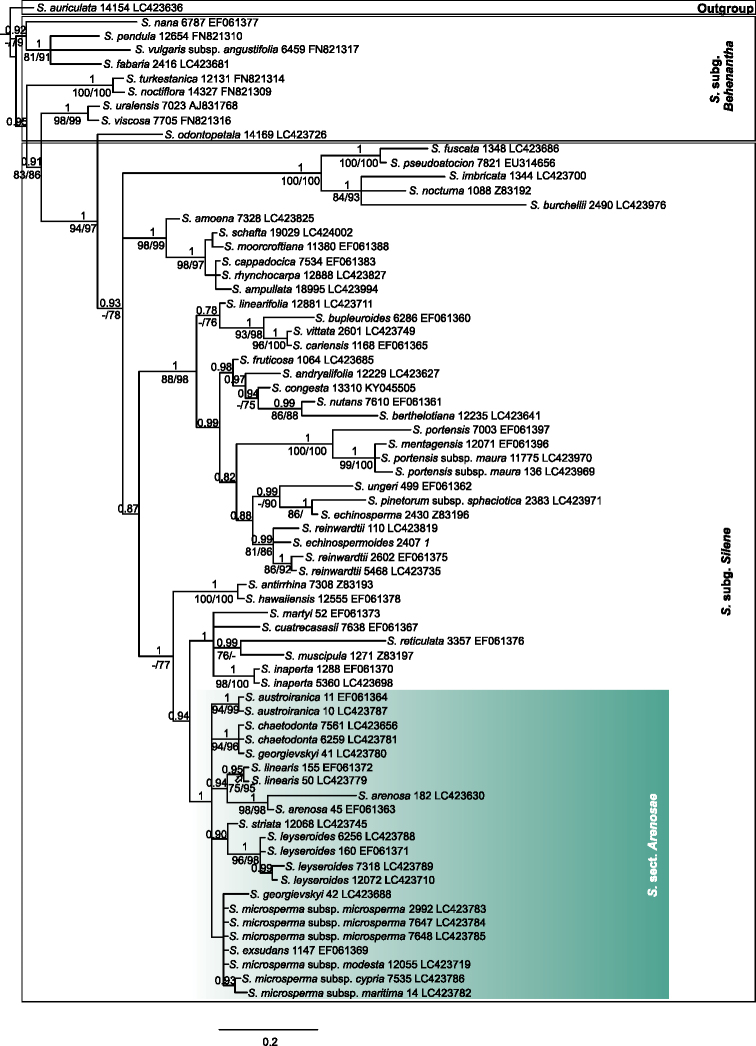
Phylogenetic tree resulting from Bayesian analysis of the *rps16* sequences including 71 taxa. The trees were summarized in a 50% majority-rule consensus tree with the posterior probabilities (PP) indicated above branches. Bootstrap support values (>75%) based on MP and ML are noted below branches, respectively. The numbers following the taxonomic name indicate the specimen ID and Genbank numbers (Suppl. material 1), respectively.

**Figure 5.**
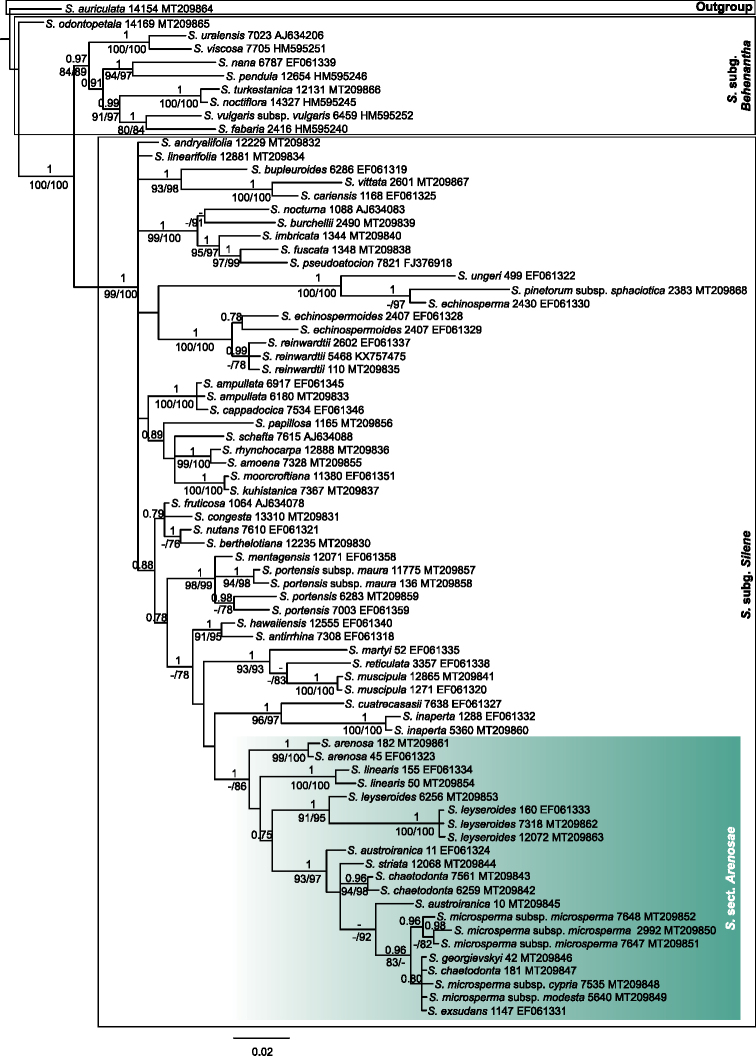
Phylogenetic tree resulting from Bayesian analysis of the *RPB2* sequences including 76 taxa. The trees were summarized in a 50% majority-rule consensus tree with the posterior probabilities (PP) indicated above branches. Bootstrap support values (>75%) based on MP and ML are noted below branches, respectively. The numbers following the taxonomic name indicate the specimen ID and Genbank numbers (Suppl. material 1), respectively.

The similarity matrix (Fig. 2) depicts pairwise posterior probabilities that different accessions cluster at approximately zero node heights. In other words, the different accessions of *S. arenosa*, *S. austroiranica*, *S. chaetodonta* Boiss., *S. leyseroides*, and *S. linearis* form distinct clusters with high support. The different accessions of *S. microsperma* Fenzl are supported moderately. The monophyly of each of the aforementioned species is also supported by the gene trees (Figs 3–5). The two accessions of *S. georgievskyi* Lazkov do not form a clade (Fig. 2): one specimen with ID 41 groups with high posterior support with the two accessions of *S. chaetodonta* in contrast to another specimen with ID 42 which with low posterior support groups with *S. microsperma*.

Silene sect. Arenosae is supported as monophyletic in the gene trees of the separate regions (PP = 1.00, *rps16*, Fig. 4; PP = 1.00, MLB = 86%, *RPB2*, Fig. 5) except in the ITS tree (Fig. 3) where the section is unresolved in relation to sect. Portenses F.Jafari & Oxelman.

## Discussion

Consistent with previous studies (Oxelman and Lidén 1995, Oxelman 1996, Eggens et al. 2007, Jafari et al. 2020), our results reveal that S. sect. Rigidulae s.l. as circumscribed by previous taxonomists from Boissier (1867) to Chater et al. (1993) is not a natural group. This broad circumscription is currently divided into five lineages (Jafari et al. 2020). Here, we concentrate on S. sect. Arenosae, which we formally describe as a new section. A taxonomic treatment and discussion of other components of S. sect. Rigidulae s.l. can be found in Jafari et al. (2020) in which lineages 1–5 refer to S. sects. *Rigidulae* s.l., *Portenses* F.Jafari & Oxelman, *Arenosae*, *Muscipula* and *Sclerocalycinae* s.l., respectively.

The use of narrow delimitations of sections has the potential to better account for the levels and patterns of diversity observed in large genera such as *Silene*, since smaller and more homogeneous groups can be circumscribed more readily, are more often geographically coherent, and are more likely monophyletic compared to larger and more heterogeneous groups. In addition, such an approach facilitates adequate or complete taxon sampling for global infrageneric studies as well as for more in-depth investigations within sections. Such an approach was successfully applied by Oxelman (1995) when he described S. sect. Sedoides Oxelman & Greuter. However, as noted by Jafari et al. (2020) the recognition of narrow groups depends on a solid understanding of the associated morphological variation, as well as on phylogenetic data from more than a couple of genetic loci (i.e., the widely used ITS and cpDNA regions).

### Morphological remarks

**Figure 6.**
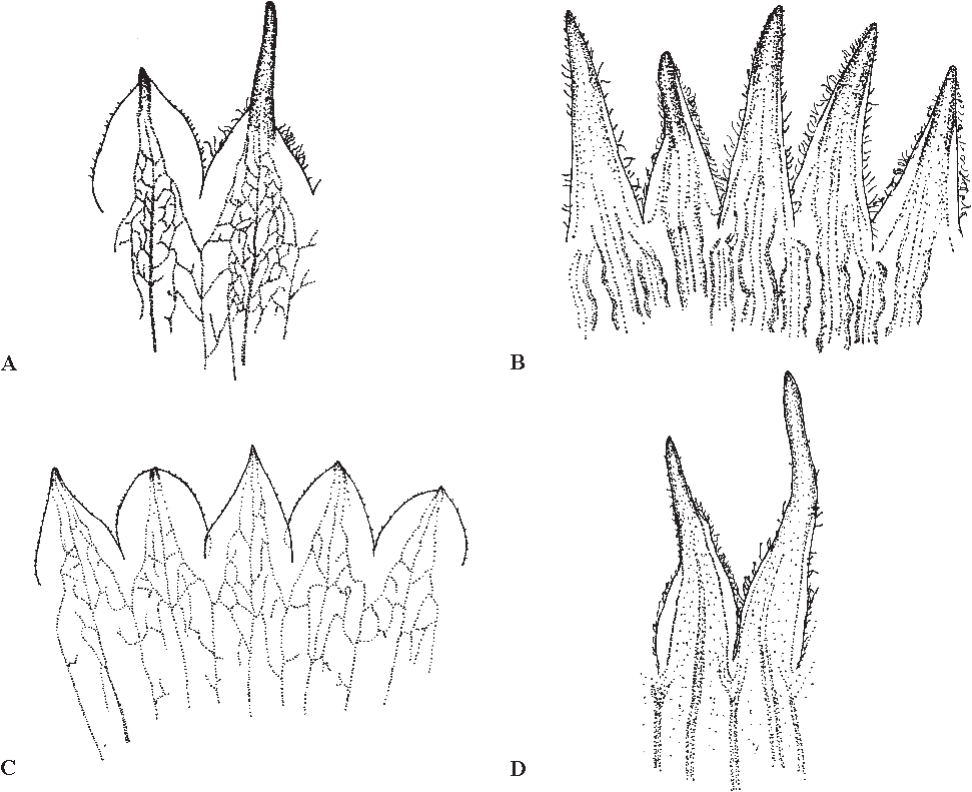
Different types of calyx teeth. **A***Silene linearis* (M. Bierkamp & P. Zinth 177 BSB) **B***Silene austroiranica* (Rechinger 10772 B) **C***Silene corinthiaca* (B. Oxelman 1934 GB) **D***Silene georgievskyi* (Rechinger 9828 B). **A**, **B** and **D** are representatives of S. section Arenosae. Illustrations by F. Eggens.

Although it is difficult to ultimately diagnose S. section Arenosae morphologically, some characters can be used to separate these species from other species of *Silene*. Contrary to its closest relatives, the basal leaves in S. section Arenosae are not spathulate, but instead oblanceolate or lanceolate. The calyx teeth in this section are usually narrowly lanceolate, terminate in a mucro and have a narrow, often densely ciliate margin. *Silene austroiranica* and *S. georgievskyi* are typical examples of species with this kind of teeth (Fig. 6B, D). By contrast, *S. corinthiaca* Boiss. (Fig. 6C), the type species of S. sect. Rigidulae (Greuter 1995), is similar to most other *Silene* spp. that have a broad transparent margin at their rounded, broadly ovate or almost deltoid calyx teeth which are narrower (at base almost as wide as long) in other components of the former S. sect. Rigidulae. *Silene linearis* (Fig. 6A) has a broad transparent calyx tooth margin, which is unique in the section, and distinct mucro, at least on three out of five calyx teeth.

The calyx teeth in *Silene* are more or less heteromorphic, with three of the five teeth different from the remaining two. They may differ in length, width, outline of the membranous margin, and ciliation (see Fig. 6). This heteromorphism is often not taken into account and only one type of tooth is described, which of course is unfortunate, especially when the heteromorphism is prominent. A few Floras (e.g. Oxelman and Greuter 1997, Chamberlain 1996) make some occasional notes on calyx tooth heteromorphism, but Maire (1963) is an exception in having clear references to three teeth having one appearance and two teeth showing another feature. In S. sect. Arenosae, the heteromorphism is primarily seen as length difference, which is easiest to spot in flower buds. We chose to use the term lanceolate (or ovate when the teeth are broad) to describe the calyx teeth instead of triangular (or broadly triangular), to emphasise the fact that the teeth are widening slightly above the base and then tapering to the apex. The green, middle part of the teeth is always triangular in outline, with slightly concave sides.

“Cauline leaves” refer to the mostly linear or lanceolate leaves on the stem, placed at least a few (3–5) cm up on the stem, as opposed to the rosulate leaves found on the lowermost parts of the stem. Coronal scales are small structures on the petals placed at the junction of the claw and limb. In most cases there are two scales that may be dentate, crenate or lacerate.

Information about the flower colors was extracted from the notes on herbarium labels or based on field or cultivation experience. *Silene* flowers in general are of two types depending on what time of the day the flowers are open to pollinators. The night-flowering flowers usually have petal limb upper surfaces being white or pale pink often with purple or greenish dorsal side with long, narrowly linear petal lobes that are typically curled up in daytime. The day-flowering flowers usually have pinkish petal limbs with entire or emarginate apices or, if the limb is bilobed, with obovate, elliptic, oblong or linear lobes. “lobes ovate” refers to petal limbs cleft less than the middle, while “lobes oblong or lobes linear” refer to petal limbs cleft to the middle or more. The day-flowering species in S. sect. Arenosae all have bilobed petal limbs. However, the majority of species are most likely night-flowering.

Many species of *Silene* may have both hermaphroditic and female flowers. The female flowers have shorter anthophores and shorter calyces, and the male organs are missing or present as rudimentary structures. The gynoecium is instead often larger. The measurements in the key and the descriptions are all based on hermaphroditic flowers.

The inflorescence in members of S. sect. Arenosae, as in many other Caryophyllaceae, is a terminal, compound dichasium accompanied by one to several axillary compound dichasia produced later. In S. sect. Arenosae, like in most species previously classified in S. sect. Rigidulae, it is often difficult to distinguish the terminal inflorescences from the lateral ones, because the axillary inflorescences from upper leaf axils are often produced almost simultaneously with the terminal ones. Pedicel length is a useful character, but has to be treated with caution, as pedicels grow through the lifespan of the inflorescence, and becomes smaller the higher up in the compound dichasium the flower is. Therefore, we only give measurements for the first flower in the terminal inforescence, both in flower and in fruit. If it is difficult to locate; one may simply look for the longest pedicel on the plant.

The species included in our study are most often puberulous or sometimes tomentose, with unicellular trichomes just barely visible with the naked eye (making the plant look greyish), or rarely villous. For all species, both leaves and stem tend to be more pubescent towards the base of the plant. Leaves are also more pubescent towards the base of each leaf, often with longer cilia at the basal leaf margin, while the leaves are often glabrous towards the apex and sometimes at the upper side. Calyces are often puberulous or tomentose when flowers are in bud, but can become almost glabrous when the fruits have developed, except on the calyx teeth. The pubescence of the calyx is often concentrated to the upper part.

#### Silene section Arenosae Eggens, F.Jafari & Oxelman, sect. nov.

Taxon classificationPlantaeCaryophyllalesCaryophyllaceae

540292E6-CA2D-5E1C-B658-0F3EF84E5317

urn:lsid:ipni.org:names:77211376-1

##### Type.

*Silene arenosa* K. Koch.

##### Description.

Annuals. Stems erect or ascending, 5–70 cm, often pubescent at least below, internodes often with sessile glands on upper part. Basal leaves lanceolate to oblanceolate, ± covered with unicellular trichomes; cauline leaves linear, lanceolate or oblanceolate, pubescent. Inflorescence an apical, uneven dichasium with long internodes, several later axillary inflorescences from upper stem nodes usually present. Flowers usually nocturnal (e.g. *S. austroiranica*, *S. linearis*), rarely diurnal (*S. exsudans* Boiss. & Heldr., *S. leyseroides*, S. microsperma subsp. cypria Eggens, F.Jafari & Oxelman, nom nov.). Calyx teeth often with distinct mucro, heteromorphic with three longer, often acute, narrowly lanceolate teeth with a narrow transparent margin, the other two teeth shorter, slightly broader, rounded and with a broad transparent margin; margin usually densely ciliate. Primary calyx veins mostly green (or reddish when exposed), often raised; secondary veins obscure; area between veins whitish. Styles 3. Petal limb upper surfaces white or pink. Capsule ellipsoid, oblong or obovate. Seeds reniform, hilum sunken, side flat, with a dorsal groove, testa smooth or papillate.

##### Distribution and habitat.

**Figure 7.**
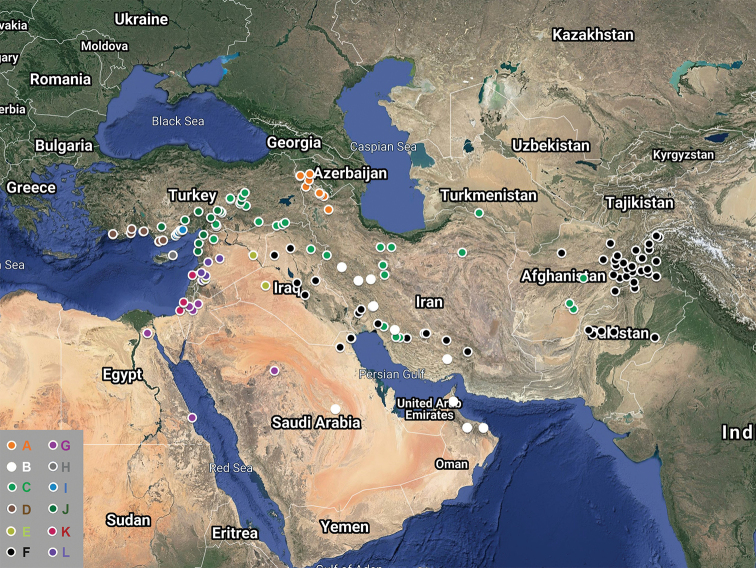
Distribution map of S. sect. Arenosae. Each color code corresponds to one taxon: **A***S. arenosa***B***S. austroiranica***C***S. chaetodonta***D***S. exsudans***E***S. georgievskyi***F***S. leyseroides***G***S. linearis***H**S. microsperma subsp. cypria**I**S. microsperma subsp. maritima**J**S. microsperma subsp. microsperma**K**S. microsperma subsp. modesta**L***S. striata*.

SW Asian, from South Mediterranean Turkey to Armenia southward to Egypt and the Arabian Peninsula and eastward to Pakistan (Fig. 7). Most taxa have rather limited distributions, except *S. chaetodonta* and *S. leyseroides* that are found from South-Central Turkey to Afghanistan and from Iraq to Pakistan, respectively. All species grow in dry sandy or gravelly habitats.

##### Notes.

Melzheimer (1988) considers *S. rhadinocalyx* Stapf [in Akad. Wiss. Wien, Math.-Naturwiss. Kl., Denkschr. 51: 352 (1886)] to belong to this group, but examination of the type led us to conclude that this taxon is closer to either of the SW Anatolian species *S. cariensis* Boiss. or *S. vittata* Stapf.

### Key to species of Arenosae

This key is most applicable to adult plants in full flower or in fruiting stage.

**Table:** 

1	Flowers diurnal; petal limbs cleft less than the middle, pink on upper surface; calyx < 10 mm; distribution: Coastal Southern Turkey	**9. *S. exsudans***
–	Flowers usually nocturnal; petal limbs cleft to the middle or more, white or pale pink on upper-surface; calyx usually >10 mm	**2**
2	Anthophore > 6 mm	**3**
–	Anthophore < 6 mm (if more, then pedicel geniculate at apex in fruit)	**5**
3	Calyx > 20 mm, longer teeth lanceolate; anthophore 13–16 mm, petal limbs 7–9 mm	**5. *S. georgievskyi***
–	Calyx < 20 mm, longer teeth ovate or lanceolate; anthophore 6.5–11 mm, petal limbs 5–8 mm	**4**
4	Calyx teeth with narrow transparent margin (cf. Fig. 6B); anthophore densely tomentose; capsule oblong	**4. *S. austroiranica***
–	Calyx teeth with broad, rounded transparent margin (cf. Fig. 6A); anthophore densely puberulent; capsule ovoid	**3. *S. linearis***
5	Calyx teeth clearly dimorphic, longer ones > 4 mm, calyx > 13 mm	**6. *S. chaetodonta***
–	Calyx teeth obscurely dimorphic, longer ones < 4 mm, calyx usually < 13 mm	**6**
6	Anthophore < 4 mm, much shorter (3 times shorter) than capsule	**8d. S. microsperma subsp. modesta**
–	Anthophore > 4 mm, slightly shorter than the capsule	**7**
7	Distinct stem internodes > 8	**8**
–	Distinct stem internodes < 8	**9**
8	Uppermost stem internode equal to the next upper one; calyx teeth 1.5–2 mm; anthophore 5–6 mm	**8b. S. microsperma subsp. cypria**
–	Uppermost stem internode clearly longer than the next upper one; calyx teeth 2–4 mm; anthophore 3–5 mm	**8a. S. microsperma subsp. microsperma**
9	Distinct stem internodes > 5; leaves fleshy	**8c. S. microsperma subsp. maritima**
–	Distinct stem internodes < 5; leaves not fleshy	**10**
10	Calyx with small papillae, the teeth ovate; anthophore glabrous; distribution: Armenia, Azerbaijan (Nachitchevan), NW Iran	**1. *S. arenosa***
–	Calyx glabrous or pubescent, but not papillate, the teeth lanceolate; anthophore puberulent to densely puberulent	**11**
11	Inflorescence divaricate, branch axile usually > 90°, pedicel geniculate, rarely erect at apex in fruit. Widespread in SW Asia	**2. *S. leyseroides***
–	Inflorescence non-divaricate, branch axile (much) less than 90°, pedicel non-geniculate at apex in fruit. Syria, Lebanon	**7. *S. striata***

#### 1. Silene arenosa

Taxon classificationPlantaeCaryophyllalesCaryophyllaceae

K.Koch, Linnaea 15: 711. 1841.

032D319C-0B0E-5C74-BE7A-11EA1833D99A

= Silene kowalenskyi Stschegl., Bull. Soc. Nat. Mosc. 26: 322. 1853. – Type: Tab. V.f.1. (neotype designated here: [Azerbaijan] Inter Nachitschevan et Ordubad, Kowalensky s.n. G-BOIS! [G00544651])

##### Type.

[Azerbaijan], Prope flumen Araxin in arena frequenter, [1837, 1838], *K.Koch 873* (lectotype, designated by Lazkov in Caucasian Flora conspectus 3(2): 208. 2012, LE! [LE01051368]; syntypes: [Azerbaijan], Araxon, annu 1838, LE! [LE01051369]; B destroyed?).

##### Description.

(5.0–)10.0–30.0 cm tall, spreading or rarely erect. Stem papillate throughout, pubescent in lower part, glabrous but with sessile glands in upper part; with 2–3 distinct internodes, the uppermost internode1.5–4.0 cm long and obviously longer than the next upper internode. Basal leaves oblanceolate, glabrous. Cauline leaves linear or lanceolate 10.0–40.0 × 2.0–4.0 mm, glabrous or slightly papillate. Calyx 10.0–14.0 mm long, cylindrical at anthesis and clavate in fruit, glabrous, slightly papillate; teeth unequal; shorter ones 1.0–1.5 mm long, ovate, mucronate; longer ones 1.5–2.0 mm, ovate, acuminate; marginal hairs short (up to 0.5 mm), sparse. Inflorescence non-divaricate, branch axile (much) less than 90°. Petal claws 6.0–7.5 mm long, glabrous; limbs 2.0–3.0 mm long, emarginate or bifid, upper-surface pink, lobes linear, petal limbs cleft to middle or more; coronal scales 0.4–0.5 mm long, ovate, apex entire. Anthophore 4.0–5.0 mm long, glabrous. Anthers exserted; filaments 7.0–8.0 mm long, glabrous. Styles exserted. First pedicel 1.0–3.0 cm in flower, 2.0–3.5 cm in fruit, spreading, glabrous, apex mostly geniculate or antrorse. Capsule 6.0–8.0 mm long, oblong or ellipsoid, fragile, opaque. Seeds 0.5–0.8 mm wide, 0.5–0.7 mm high, testa smooth.

##### Distribution.

Armenia, Azerbaijan (Nachitchevan), NW Iran (Fig. 7).

##### Notes.

The two accessions form a strongly supported clade in all trees (PP = 1.00, Fig. 1; PP = 1.00 MPB = 100% MLB = 100%, Fig. 3; PP = 1.00 MPB = 98% MLB = 98%, Fig. 4; PP = 1.00 MPB = 99% MLB = 100%, Fig. 5). Despite its geographical, morphological and phylogenetic distinctiveness, this taxon has been confused with *S. leyseroides* (Melzheimer 1988: as synonym, Schischkin 1936). The two species are superficially similar; both have spreading stems and pedicels that are upturned (or geniculate) at apex in fruit, so that all capsules are vertical although the pedicel may be almost horizontal. However, *S. arenosa* is readily distinguished by the shorter, mucronate and sparsely ciliate (not acuminate and densely ciliate) calyx teeth and the glabrous anthophore from *S. leyseroides*. It also has smaller petals that are almost completely included within the calyx, and the petal limb is sometimes emarginate rather than bilobed. We have not seen any material of *S. arenosa* from any other area than Armenia, Azerbaijan (more specifically the region Nachitchevan), and Iran (close to the borders to Armenia, Turkey, and Nachitchevan), whereas *S. leyseroides* appears to be allopatric and grows mainly in the Zagros Mountain range and in E Afghanistan/NW Pakistan (see Fig. 7).

The seeds of *S. arenosa* are possibly more shining on the surface, instead of the greyish, dull surface that is the common condition for *Silene* seeds, but we have seen too few specimens with seeds to draw definitive conclusions. The green midpart of the calyx teeth is narrow, which can make the teeth look lanceolate rather than ovate. Collections from near the border between Iran and Turkey have calyces which are densely papillose in upper parts.

#### 2. Silene leyseroides

Taxon classificationPlantaeCaryophyllalesCaryophyllaceae

Boiss., Diagn. Pl. Orient. 1:41. 1843.

E5505054-82BE-5B77-A40C-087A5BC448F5

= Silene salsa Boiss., Diagn. Pl. Orient. 8:77. 1849. – Type: [Iran], Hab. in solo salso ad lacum Nemek Derja prope Schiras, 1 April 1842, *K.G.T. Kotschy*, *pl. Pers. austr. 453* (lectotype, designated here: G-BOIS! [G00544649], isolectotypes: G! [G00226818, G00226819, G00226820], C! [C10009174, C10009175], K! [K000728456], WAG! [WAG0191878])

##### Type.

[Iraq], Hab. ad Babylonem [in deserto Babylonia], *Aucher Eloy*, *pl. exs. 448* (lectotype, designated here: G-BOIS! [G00544647]; isolectotypes: G! [G00226728, G00226729], K! [K000728455]).

##### Description.

5.0–35.0 cm tall, spreading or rarely erect. Stem pubescent in lower part, more or less glabrous with sessile glands in upper part; with 3–5 distinct internodes, the uppermost internode (1.0–)2.0–3.0(–4.0) cm long and obviously longer than the next upper internode. Basal leaves oblanceolate or lanceolate 10.0–30.0 × 1.0–3.0 mm, pubescent, scabrous. Cauline leaves linear or lanceolate 20.0–35.0 × 2.0–3.0 mm, pubescent, scabrous. Calyx (8.0–)9.0–13.0(–14.0) mm long, cylindrical at anthesis and clavate in fruit, rarely glabrous, or pubescent; teeth unequal; shorter ones 1.0–2.0 mm, lanceolate, acuminate; longer ones 2.0–3.0(–4.0) mm, lanceolate, acuminate; marginal hairs long (longer than 0.5 mm), dense. Inflorescence divaricate, branch axile usually > 90°. Petal claws 6.0–7.0 mm long, glabrous; limbs 4.0–7.0 mm long, bifid, upper-surface pink, lobes linear, divergent, petal limbs cleft to middle or more, lower-surface carmine or green; coronal scales 0.8–1.1 mm long, ovate, apex entire or slightly dentate. Anthophore (4.0–) 5.0–7.0 mm long, densely puberulent. Anthers exserted; filaments 7.0–8.0 mm long, glabrous. Styles exserted. First pedicel 1.0–3.0 cm in flower, 2.0–4.0 cm in fruit, spreading, glabrous, apex usually geniculate, or antrorse. Capsule 6.0–8.0 mm long, oblong or ellipsoid, fragile, opaque. Seeds 0.6–0.9 mm wide, 0.4–0.6 mm high, testa smooth.

##### Distribution.

Iraq, Iran, Kuwait, Afghanistan and Pakistan (mainly in the Zagros range of Iran and in E Afganistan/NW Pakistan) (Fig. 7).

##### Notes.

This species is recognized by a spreading growth form with many branches from the base, upturned (or geniculate) pedicels at apex in fruit and narrowly lanceolate calyx teeth. The calyx veins are often reddish or purplish in dried material (probably green in fresh state). The petal lobes are linear and divergent.

The specimens from the eastern parts of the distribution area tend to have less pubescent calyces (sparsely puberulous or almost glabrous) and are less pubescent on stem and leaves. However, a specimen from NE Saudi Arabia (Mandaville 1645 BM) is almost glabrous on calyces and puberulous on stem and leaves.

From the original description, *S. cabulica* Bornm. [in Engl. Jahrb. 46, 221–222 (1934), type from around Kabul) seems to be very similar to *S. leyseroides*. We have, however, not been able to trace any type material and propose that the type was destroyed in B. Both Ghazanfar and Nasir (1986) and Melzheimer (1988) mention *S. cabulica* as dubious.

The *S. leyseroides* clade is strongly supported (PP = 1.00, Fig. 1; PP = 1.00 MPB = 100%, MLB = 100%, Fig. 3; PP = 1.00 MPB = 96% MLB = 98%, Fig. 4; PP = 1.00 MPB = 91% MLB = 95%, Fig. 5). Three of the *S. leyseroides RPB2* sequences (from Iran, Iraq and Kuwait) share a unique 252 bp insertion. Interestingly, this insertion is not found in the specimen from Afghanistan. The accessions from Iran, Iraq and Kuwait form a strongly supported clade (PP = 1.00 MPB = 100%, MLB = 100% Fig. 5).

#### 3. Silene linearis

Taxon classificationPlantaeCaryophyllalesCaryophyllaceae

Decne., Ann. Sci. Nat. Bot. sér. 2, 3: 276. 1835, nom. cons. prop. (in press) [non Sweet].

EB490462-58C3-5C76-A38A-E749F0598BDD

##### Type.

[Egypt], Hab. le désert du Sinaï, [1.6.1832], *N. Bové 178* (lectotype, designated here: G! [G00226732]; isolectotypes: K! [K000728452], G! [G00226733]).

##### Description.

15.0–60.0 cm tall, erect or spreading. Stem pubescent in lower part, scabrous, glabrous but with sessile glands in upper part; with 6–10 distinct internodes, the uppermost internode length 3.0–6.0 cm long and obviously longer than the next upper internode. Basal leaves oblanceolate 30.0–60.0 × 2.0–4.0 mm, pubescent. Cauline leaves linear or lanceolate 10.0–55.0 × 1.0–4.0 mm, pubescent. Calyx 11.0–19.0 mm long, campanulate at anthesis and clavate in fruit, pubescent; teeth unequal; shorter ones 1.5–2.0 mm, ovate, mucronate; longer ones 2.0–2.5 mm, ovate, acuminate; marginal hairs short (up to 0.5 mm), dense. Inflorescence non-divaricate, branch axile (much) less than 90°. Petal claws 6.0–7.0 mm long, glabrous; limbs 6.0–8.0 mm long, divided, upper-surface white, lobes linear or oblong, divergent, petal limbs cleft to middle or more, lower-surface green; coronal scales 1.0–2.5 mm long, obovate, apex dentate. Anthophore 8.0–11.0 mm long, densely puberulent. Anthers exserted; filaments 8.0–9.0 mm long, glabrous . Styles exserted. First pedicel 1.0–3.0 cm in flower, 2.0–4.0 cm in fruit, erect, glabrous, apex antrorse. Capsule 5.0–7.0 mm long, ovoid or ellipsoid, fragile, opaque. Seeds 0.7–0.9 mm wide, 0.6–0.7 mm high, testa smooth.

##### Distribution.

E Egypt (Red Sea area, Sinai), N Arabian Peninsula, W Jordan and Palestine (Fig. 7).

##### Notes.

*Silene linearis* has some superficial similarity to *S. austroiranica*, which has narrowly lanceolate calyx teeth with narrow transparent margin, and not the broad rounded margin of *S. linearis* (see Fig. 6). *Silene austroiranica* is allopatric and found further south and east on the Arabian Peninsula, and in eastern Iraq and western/southern Iran.

The ranges of the calyx, anthophore and capsule lengths are unusually large in *S. linearis*. The large-flowered individuals are all found in Egypt (although not all specimens from Egypt are large-flowered), with calyx length of 17–19 mm (and proportional anthophores and capsules). The specimens are in all other respects similar (or perhaps with slightly shorter mucro on calyx teeth) to the *S. linearis* specimens with smaller flowers, and we do not think the difference is sufficient to merit taxonomic recognition. The Egyptian specimens are in general (independent of flower size) tomentose to villous while the specimens from Palestine and Jordan are often slightly puberulous, although at least one specimen from Palestine is densely tomentose.

One sequence for a specimen from Egypt (*S. linearis*, ID 49, KX757593) is included in the ITS tree. It forms a strongly supported clade together with the other two *S. linearis* accessions (PP = 1.00 MPB = 96% MLB = 93%, Fig. 3). The *S. linearis* clade (with the two Palestine accessions) is strongly supported in all trees (PP = 1.00, Fig. 1; PP = 0.95 MPB = 75% MLB = 95%, Fig. 4; PP = 1.00 MPB = 100% MLB = 100%, Fig. 5).

##### Nomenclatural notes.

The name *Silene linearis* Decne. has been used for a long time, but the delimitation of the taxon has varied. A number of authors have used the name in our sense, e.g. Boissier (1867), Rohrbach (1868), Williams (1896), Post (1932), Chowdhuri (1957), Mouterde (1966), Zohary (1966), Chamberlain (1996) and Boulos (1999). Other authors use this name for a more inclusive taxon, e.g. Rechinger (1964) and Blakelock (1957), including *S. leyseroides*, *S. arenosa*, *S. chaetodonta* and *S. kotschyi* Boiss. (= *S. microsperma*). Sweet (1830) used the epithet “*linearis*” in Hortus Britannicus 2^nd^ ed., in a completely different context, five years earlier than Decaisne’s description was published. The name *Silene linearis* Sweet has been cited by few authors. Rohrbach (1868) referred to the name as a synonym for *Silene cucubalus* Wib. (= *Silene vulgaris* (Moench) Garcke) and Marsden-Jones and Turrill (1957) recognized the name as a part of the *Silene vulgaris*-assemblage but used the name in a highly informal way. The name is not mentioned in Chater et al. (1993), Aeschimann (1985), Pignatti (1982) or Greuter et al. (1984). *Silene linearis* Decne. has been suggested to be conserved against *Silene linearis* Sweet (Eggens & al., in press).

#### 4. S. austroiranica

Taxon classificationPlantaeCaryophyllalesCaryophyllaceae

Rech.f., Aell. & Esfand., Bot. Jahrb. Syst. 75: 349. 1951.

9A620608-3D84-56E6-9C31-A74D4923C73B

##### Type.

[Iran], Lar. [Hormozgan] Hadjiabad prope Tarum, ca. 900 m, 29 April 1948, *K.H. Rechinger*, *P. Aellen & E. Esfandiari 3386* (holotype: W! [W19800014919]; isotypes: G! [G00006016, G00006017], S! [S-G-8718]).

##### Description.

15.0–50.0 cm tall, erect. Stem pubescent in lower part, pubescent in upper part; with 3–5 distinct internodes, the uppermost internode 1.0–10.0 cm long and obviously longer than the next upper internode. Basal leaves oblanceolate 10.0–30.0 × 1.0–6.0 mm, pubescent. Cauline leaves oblanceolate 5.0–40.0 × 2.0–6.0 mm, pubescent. Calyx 12.0–16.0 mm long, campanulate at anthesis and clavate in fruit, glabrous or pubescent; teeth unequal; shorter ones 2.0–3.0 mm, ovate, acuminate; longer ones 2.0–4.0 mm, lanceolate, acuminate; marginal hairs long (longer than 0.5 mm). Inflorescence non-divaricate, branch axile (much) less than 90°. Petal claws 7.0–10.0 mm long, glabrous; limbs 5.0–6.0 mm long, divided, upper-surface white or pink, lobes linear, divergent, petal limbs cleft to middle or more; coronal scales 1.3–2.0 mm long, elliptic or obovate, apex slightly dentate. Anthophore 6.5–9.0 mm long, densely tomentose. Anthers exserted; filaments 8.0–12.0 mm long, glabrous. Styles exserted. First pedicel 1.0–3.0 cm in flower, 2.0–5.0 cm in fruit, erect, glabrous, apex antrorse. Capsule 5.5–8.0 mm long, oblong or ellipsoid, fragile, translucent. Seeds 0.5–0.8 mm wide, 0.5–0.7 mm high, testa smooth.

##### Distribution.

Arabian Peninsula, Kuwait, Iraq and Iran (Fig. 7).

##### Notes.

This species has rather long internodes, two to ten times the length of the subtending leaves (rarely of the same length). In particular, the uppermost internode is long, sometimes as long as 10 cm. Plants from the Riyadh area tend to have shorter upper internodes. The internodes are often viscid. The long internodes together with the relatively long coronal scales are the best characters for recognizing this species.

The specimens from Iran tend to have broader leaves than the other specimens, in particular the ones from the Arabian Peninsula.

The clade with the two *S. austroiranica* accessions is strongly supported in the species (PP = 1.00, Fig. 1), ITS (PP = 1.00 MPB = 85% MLB = 98%, Fig. 3) and *rps16* trees (PP = 1.00 MPB = 94% MLB = 99%, Fig. 4). The two accessions of *S. austroiranica* do not form a clade in *RPB2* tree, probably due to difference in sequence length (one accession was 490 bp and another 140 bp: due to incomplete sequence read). In the *RPB2* tree the *S. austroiranica* clade is nested within a clade including *S. microsperma*, *S. exsudans*, *S. chaetodonta*, *S. striata* Ehrenb. ex Rohrb. and *S. georgievskyi* (PP = 1.00 MPB = 93% MLB = 97%, Fig. 5), but in the ITS phylogeny *S. austroiranica* and *S. linearis* are successive sisters to this clade (PP = 0.99 MPB = 75% MLB = 78% and PP = 0.95 MPB = 85% MLB = 88%, Fig. 3).

#### 5. S. georgievskyi

Taxon classificationPlantaeCaryophyllalesCaryophyllaceae

Lazkov, Bot. Zhurn. (Moscow & Leningrad). 84 (9): 123. 1999.

5B499517-3CAE-56FE-8454-00959F67BC37

##### Type.

[Syria], Desertum Syriacum. 30 km ad austro-orient. Ab urb. Deir-Ez-Zor, vallis undulata, ass. Ephem.-car. Frequens, 15 May 1985, *A. Georgievsky s.n.* (Holotype: LE! [LE01051363]).

##### Description.

20.0–50.0 cm tall, erect. Stem pubescent in lower part, scabrous, pubescent with sessile glands in upper part; with 8–12 distinct internodes, the uppermost internode obviously longer than the next upper internode. Basal leaves linear or oblanceolate, pubescent. Cauline leaves linear 10.0–40.0× 1.0–3.0 mm, pubescent. Calyx 25.0–30.0 mm long, ovoid at anthesis and clavate in fruit, pubescent; teeth unequal; shorter ones 2.0–4.0 mm, ovate, acuminate; longer ones 4.0–6.0 mm, lanceolate, acuminate; marginal hairs long (longer than 0.5 mm), dense. Inflorescence non-divaricate, branch axile (much) less than 90°. Petal claws 10.0–12.0 mm long, glabrous; limbs 7.0–9.0 mm long, bifid, upper-surface pink, lobes oblong, petal limbs cleft to middle or more; coronal scales 2.0–2.2 mm long. Anthophore 13.0–16.0 mm long, glabrous or puberulent. Anthers exserted; filaments 12.0–15.0 mm long, glabrous. Styles exserted. First pedicel 1.0–4.0 cm in flower, 2.0–6.0 cm in fruit, erect, glabrous, apex antrorse. Capsule 12.0 mm long, oblong or ellipsoid. Seeds 0.8–1.0 mm wide.

##### Distribution.

Syria, N Iraq (Fig. 7).

##### Notes.

At the molecular level, we have two sequences for each ITS and *rps16* and only one for *RPB2*. All the three markers were sequenced for the specimen from Syria (*S. georgievskyi* ID. 42), but for the specimen from Iraq, the ITS and *rps16* regions were sequenced from two duplicate specimens from different herbaria. The two accessions of *S. georgievskyi* from Iraq and Syria do not form a monophyletic group in the species, ITS and *rps16* trees (Figs 1, 3, 4). The accession from Iraq (*S. georgievskyi* ID. 41) is found together with the accessions of *S. chaetodonta* in a moderately to strongly supported clades in the species (PP = 0.78, Fig. 1) and *rps16* (PP = 1.00 MPB = 94% MLB = 96%, Fig. 4) trees, respectively. The accession from Syria is nested within a clade including *S. microsperma* in the species tree (Fig. 1) and weakly supported in *rps16* tree (Fig. 4, PP<0.75). In the ITS tree, the accessions of *S. georgievskyi* do not form a monophyletic group, but they are included in a strongly supported clade together with *S. chaetodonta* and *S. striata* (PP = 0.98 MPB = 86% MLB = 93%, Fig. 3). The morphological distinctiveness (much longer calyx, long anthophore and larger petals) speaks in favour of recognition of the species, and although chromosome numbers are unknown, we hypothesize that the incongruent pattern seen in the Syrian specimen may be explained by polyploid hybridization. Allopolyploids often grow larger than their parents (Chen 2010). *Silene georgievskyi* is morphologically larger in floral and general habit aspects compared to both *S. chaetodonta* and *S. microsperma*. There may be a small overlap in the distributions of *S. chaetodonta* and *S. georgievskyi*, in the border area between Iraq and Syria.

#### 6. Silene chaetodonta

Taxon classificationPlantaeCaryophyllalesCaryophyllaceae

Boiss., Diagn. Pl. Orient. 1: 39. 1843.

5AEC9187-2E9C-5527-B64E-53F46A1776C4

= Silene chaetodonta Boiss. var. pittodes Boiss., Fl. Or. 1: 606. 1867. – Type: [Iran], Hab. In Persiâ ad Schurab inter Ispahan et Teheran, May 1859, *Bunge s.n.* (holotype: G-BOIS! [G00544221])
= S. debilis Stapf, Akad. Wiss. Wien, Math.-Naturwiss. Kl., Denkschr. 51: 282. 1886. – Type: [Iran], [In agro Ecbatanensi], In colle prope Hamadan, 8 June 1882, *Th. Pichler s.n. in D.J.E. Polak Iter Persicum* (lectotype, designated here: K! [K000728462]; isolectotype: G! [G00378634])

##### Type.

[Iran], Hab. In Persia australis, *Aucher Eloy Pl. Exs. 4223* (lectotype, designated by Lazkov in Bot. Zhurn. (Moscow & Leningrad). 87 (5): 130. 2002) G! [G00378632]; isolectotypes: G-BOIS! [G00544217], LE! [LE01051365], BM! [BM000990893], K! [K000728461], MO! [MO-149678]).

##### Description.

15.0–60.0 cm tall, erect or rarely spreading. Stem pubescent in lower part, scabrous, glabrous but with sessile glands in upper part; with 4–12 distinct internodes, the uppermost internode (2.0–)3.0–8.0(–10.0) cm long and obviously longer than the next upper internode. Basal leaves oblanceolate, pubescent. Cauline leaves linear or oblanceolate 10.0–50.0 × 2.0–6.0 mm, pubescent, scabrous. Calyx 13.0–17.0 mm long, ovoid at anthesis and clavate in fruit, scabrous; teeth unequal; shorter ones 2.0–4.0 mm, lanceolate, acuminate; longer ones 4.0–7.0 mm, lanceolate, acuminate; marginal hairs long (longer than 0.5 mm), dense. Inflorescence non-divaricate, branch axile (much) less than 90°. Petal claws 7.0–8.0 mm long, glabrous; limbs 5.0–8.0 mm long, bifid, upper-surface pink, lobes oblong, petal limbs cleft to middle or more; coronal scales 1.0–1.5 mm long, ovate, apex dentate. Anthophore 4.0–6.0 mm long, densely puberulent. Anthers included; filaments 8.0–9.0 mm long, glabrous. Styles exserted or included. First pedicel 1.0–4.0 cm in flower, 2.0–6.0 cm in fruit, erect, glabrous, apex antrorse. Capsule 7.0–11.0 mm long, oblong or ellipsoid, robust. Seeds ca 1.1 mm wide, ca. 0.7 mm high, testa smooth.

##### Distribution.

Iran, SE Turkey, Syria, Iraq, S Turkmenistan, Afghanistan, and NW Pakistan (Fig. 7).

##### Notes.

Usually, this species is readily distinguished by its whitish stems, pink and broad lobed petal limbs, long calyx teeth, total calyx length less than 20 mm, prominent calyx vein and thick, robust capsule wall. *Silene georgievskyi* differs from it by having a much longer calyx and anthophore. It seems that the length of the calyx teeth is a more important character than calyx total length for species delimitation in this group.

We have sequenced all selected markers for two specimens from the same geographical region (W Iraq). The *RPB2* sequences generated for two accessions of *S. chaetodonta* (ID 6259 and ID 7561) and one for *S. striata* shared a unique 261 bp insertion, but one accession of *S. chaetodonta* from Turkey (ID 181) and one of *S. georgievskyi* (ID 42: probably a hybrid between *S. chaetodonta* and *S. microsperma*, see above) lack this insertion. The two accessions of *S. chaetodonta* from W Iraq form a clade in the *RPB2* tree (PP = 0.96 MPB = 94% MLB = 98%, Fig. 5), but the accession from Turkey is not sister to this clade and is nested within a clade including *S. microsperma*, *S. exsudans* and *S. georgievskyi* ID 42 (PP = 0.96 MPB = 83%). The accession of *S. chaetodonta* from Turkey could be a hybrid between *S. chaetodonta* and *S. microsperma* according to *RPB2* sequence analysis. An accession from NE Iran (*S. chaetodonta* ID 7642) form a clade with the other two *S. chaetodonta* sequences in the ITS tree (PP = 0.99 MPB 86% MLB = 90%, Fig. 3). The accession from NE Iran generated only an ITS sequence in our analyses.

#### 7. Silene striata

Taxon classificationPlantaeCaryophyllalesCaryophyllaceae

Ehrenb. ex Rohrb., Bot. Zeitung (Berlin) 25: 83. 1867.

CBA6151C-7469-5B9E-AC79-E7E290275BC8

##### Type.

[Syria], In der Ebene von Baalbek in Syrien, *C.G. Ehrenberg* (no specimen traced); (neotype, designated here: [Syria] Antiliban, entre la Sahara et Dimas (Al-Dimas), 9 June 1868, *C. Gaillardot 1643* as *S. kotschyi* G-BOIS! [G00544635]).

##### Description.

10.0–20.0 cm tall, erect. Stem with sessile glands in central and upper parts; with 3–5 distinct internodes. Cauline leaves linear 20.0 × 2.0 mm. Calyx 12.0–13.0 mm long, campanulate at anthesis and clavate in fruit, glabrous or sparsely pubescent; teeth unequal; shorter ones 1.0–1.5 mm, lanceolate, acuminate; longer ones 2.0–3.5 mm, lanceolate, acuminate; marginal hairs long (longer than 0.5 mm), dense. Inflorescence non-divaricate, branch axile (much) less than 90°. Petal claws 6.0–6.5 mm long, ciliate; limbs 6.0 mm long, bifid to less than half, upper-surface pink, lobes oblong, petal limbs cleft to middle or more, divergent; coronal scales 2.0 mm long, ovate, apex entire. Anthophore ca 5.5 mm long, puberulent. Anthers exserted; filaments glabrous. Styles exserted. First pedicel 1–2 cm in flower, 2–3 cm in fruit, erect or spreading, apex antrorse. Capsule 6.0–8.0 mm, oblong, fragile, opaque. Seeds unknown.

##### Distribution.

Syria, Lebanon (Fig. 7).

##### Notes.

This species is distinguished by its small size, rather short calyx (12–13 mm) and calyx teeth (2–3.5 mm), oblong or slightly obovate petal lobes and ciliate petal claws, and strongly exserted anthers and styles.

The sequences from the three different markers analyzed here are incongruently positioned in the phylogenies. In the ITS tree, this species is found in a clade including *S. georgievskyi* and *S. chaetodonta*, as sister to the latter but with moderate support (PP = 0.80, Fig. 3). It is unresolved in a relatively large clade in the *RPB2* tree, although shares a 261 bp insertion with the *S. chaetodonta* accessions (*S. georgievskyi* sequence is missing for this marker). In the *rps16* tree, *S. striata* is sister to the *S. leyseroides* clade (PP = 0.90, Fig. 4). Morphology, geographical distribution and other molecular characteristics (e.g. the long insertion shared by *S. striata* and *S. chaetodonta*) suggest that *S. striata* is more closely related to *S. chaetodonta* than *S. leyseroides*.

#### 8. Silene microsperma

Taxon classificationPlantaeCaryophyllalesCaryophyllaceae

Fenzl, Pug. Pl. Nov. Syr. 9. 1842.

30903FB0-1B54-5FDB-8A56-76EE140AEF9A

##### Type.

See below subspecies.

##### Distribution.

Turkey, Syria, N Iraq, Cyprus, Palestine and Lebanon (Fig. 7).

##### Notes.

This species is the most variable in the section and is here divided into four subspecies. We have chosen not to treat these taxa as species because they are obviously closely related, as seen by low variation in the DNA sequences. The taxon “*S. modesta*” has sometimes been treated as a species (e.g. Zohary 1966, Mouterde 1966), but has also previously been treated as a variety of *S. chaetodonta* (Post 1932). Here, we accept it as a subspecies of *S. microsperma*.

The *S. microsperma* accessions with *S. exsudans* and one accession of *S. georgievskyi* ID. 42 form a weakly supported clade in the species (Fig. 1) and *rps16* (PP < 0.75) trees. The *RPB2* tree shows almost the same pattern, but *S. chaetodonta* ID 181 from Turkey is included in this clade (PP = 0.96 MPB = 83%, Fig. 5). The ITS phylogeny supports a close relationship between *S. microsperma* and *S. exsudans* (PP = 0.98 MPB = 86% MLB = 97%, Fig. 3). There is very little resolution within the *S. microsperma* clade.

#### 8a. Silene microsperma subsp. microsperma

Taxon classificationPlantaeCaryophyllalesCaryophyllaceae

.

F7461C70-CC06-5DB2-9AEA-ED398C52BF04

= Silene kotschyi Boiss., Diagn. Pl. Orient. 1: 40. 1843. – Type: [Turkey], In monte Tauro, [1836], *K.G.T. Kotschy 85* (lectotype, designated here: G-BOIS! [G00544619]; isolectotypes: W! [W19580022871], BM! [BM000990903], LE! [LE01051362], TUB! [No Barcode], G! [G00226928, G00226929, G00226930], KFTA [KFTA0001153]); syntypes: [Syria], Syria prope Aintab, *Aucher Eloy 425* (G! [G00226812, G00226931], G-BOIS! [G00544620], BM! [BM000990904], E! [E00286983])
= Silene kotschyi var. effusissima Boiss., Fl. Or. Suppl. 85. 1888. – Type: [Turkey], Hab. Syriæ Marasch in agris, [15.7.1865], *H.K. Haussknecht s.n.* (lectotype, designated here: G-BOIS! [G00544631]; isolectotypes: JE! [JE00013446, JE00013447]; [Iran/Iraq] In apricis calcaries m. Schahu et Avroman Kurdistaniæ, 6000’, *H.K. Haussknecht 192* (syntypes: JE! [JE00013444, JE00013445]).
= Silene cassia Boiss., Diagn. Pl. Orient. 8: 78. 1849. – Type: [Syria], Hab. in sylvaticis jugi Cassii ubi exemplaria pauca, [May-July] 1846, *P.E. Boissier s.n.* (lectotype, designated here: G-BOIS! [G00544654]; isolectotypes: G! [G00226837], LE! [LE01051366])

##### Type.

[Turkey] Prope Süveydiye, ad Orontis, *K.G.T. Kotschy s.n.* (no specimen cited).

##### Description.

15.0–70.0 cm tall, erect or spreading. Stem pubescent in lower part, scabrous, glabrous but with sessile glands in upper part; with 8–12(–20) distinct internodes, the uppermost internode (3.0–)4.0–6.0(–7.0) cm long and obviously longer than the next upper internode. Basal leaves linear or oblanceolate 1.0–4.0 × 1.0–4.0 mm, pubescent. Cauline leaves linear 10.0–30.0× 1.0–3.0 mm, pubescent. Calyx 9.0–14.0 mm long, campanulate at anthesis and clavate in fruit, pubescent; teeth unequal; shorter ones 2.0–3.0 mm, lanceolate, acuminate; longer ones 2.0–4.0 mm, lanceolate, acuminate; marginal hairs long (longer than 0.5 mm), dense. Inflorescence non-divaricate, branch axile (much) less than 90°. Petal claws 4.0–7.5 mm long, ciliate; limbs 5.0–6.5 mm long, bifid, upper-surface white or pink, lobes oblong, petal limbs cleft to middle or more; coronal scales 0.8–1.4 mm long, ovate, apex dentate or erose. Anthophore 3.0–5.0 mm long, densely puberulent. Anthers exserted; filaments 6.0–9.0 mm long, sometimes pubescent. Styles exserted. First pedicel 1.0–3.0 cm in flower, 1.0–4.0 cm in fruit, erect, glabrous, apex antrorse. Capsule 6.0–7.0 mm long, oblong, fragile, opaque. Seeds 0.6–1.0 mm wide, 0.4–0.8 mm high, testa smooth or papillate.

##### Distribution.

South Central Turkey, W and N Syria (Fig. 7). Specimens from near the border between Iraq and Iran with ciliate petal claws but in other characteristics resembling *S. chaetodonta* have been suggested to be of hybrid origin (Melzheimer 1988) and deserve closer investigation.

##### Notes.

The stem often has a larger number of internodes than other taxa in the section, sometimes as many as 20, although more often up to 12 clearly separated, distinct stem internodes. The middle internodes are shorter than or up to two (three) times the length of the subtending pair of leaves (the basalmost nodes are very short for all species). This gives this taxon a “leafy” appearance, reinforced by many branches and leafy shoots in leaf axils. The uppermost axillary branches are often opposite. This taxon is very variable, but is recognized by the many internodes, the ciliate petal claws and the small mamillae on the seeds.

*Silene cassia* is the name used for white flowered variants according to Coode and Cullen (1967). It is possible that the name *S. ehrenbergiana* Rohrb. [in Bot. Zeitung (Berlin) 25: 83. 1867. – Type: “Bei Fakra (?) in Syrien im Juni” Ehrenberg, B destroyed?] is associated with this taxon, but we have not been able to confirm this.

##### Nomenclatural notes.

Many authors have used the name *S. kotschyi* Boiss. for this species (e.g. Boissier 1867, Williams 1896, Post 1932, Chowdhuri 1957, Mouterde 1966, Coode and Cullen 1967, Meikle 1977). Melzheimer (1988) treated *S. kotschyi* Boiss. as a synonym of *S. microsperma* Fenzl. We have not been able to find any type specimen of *S. microsperma*. Fenzl noted *specimen unicum* in the protologue, so it is possible that the only type material has been destroyed during the Second World War bombings of Berlin. The description made by Fenzl is short and unspecific and fits any species in S. sect. Arenosae. However, Rohrbach (1868) used the name *S. microsperma* Fenzl and listed *S. kotschyi* Boiss. as a synonym, and it is likely that he had seen the specimen cited by Fenzl. Burtt and Lewis (1952) use the name *S. kotschyi* Boiss., but they cited the publication year as 1842, the same as for *S. microsperma* Fenzl. Stafleu and Cowan (1976) stated 1843 as the true publication year for the first part of Boissier’s *Diagnoses plantarum Orientalum novarum*. Burtt and Lewis (1952) pointed out that Rohrbach described *S. microsperma* as having glabrous petal claws, not ciliate as the taxon dealt with here. The type specimen for *S. microsperma* Fenzl was collected in an area that nowadays belongs to Turkey, at the mouth of the river Nahr al-Asi (also known as Orontis/Orontes), probably near Samandagi (old name Süveydiye, probably the same as Svedie). There are collections from this area (*Haradjian 3069* in G, *Pabot s.n.* in G, *Mouterde V 58* in G, *Haradjian 1480* in E, *Davis*, *Dodds & Cetik 19551* in C) that clearly belong to this taxon. The type locality for *S. cassia* Boiss. is also found in this area. We therefore follow Melzheimer (1988) and use the name *S. microsperma* Fenzl for this taxon.

The S. microsperma subsp. microsperma accessions form a subclade in the *S. microsperma* clade in the *RPB2* phylogeny (PP = 0.96, Fig. 5).

#### 8b. Silene microsperma subsp. cypria

Taxon classificationPlantaeCaryophyllalesCaryophyllaceae

Eggens, F.Jafari & Oxelman nom. nov.

98488E30-A50C-5A58-972B-AC1EBEB36566

urn:lsid:ipni.org:names:77211377-1

≡ Silene stenocalyx H.Lindb., Acta Soc. Sci. Fenn., Ser. B, Opera Biol. 2(7): 15. 1946. nom. illeg. [non Rouy & Foucaud]. Type: [Cyprus], Famagusta, in colle arenoso juxta mare, 8 July 1939, *H. Lindberg s.n.* (lectotype, designated by G. Lazkov in H. Väre (2012: 82): H! [H-1339014]; isolectotypes: LE! [LE01051367], H! [H-1339012, H1339013, H1339014, H1339015, H1339017], K! [K000728453, K000728454], CAI! [CAI000023])
≡ Silene kotschyi Boiss. var. stenocalyx (H. Lindb.) Chowdhuri, Notes Roy. Bot. Gard. Edinburgh 22: 276. 1957. Type: Based on S. stenocalyx

##### Description.

20.0–40.0 cm tall, erect or spreading. Stem pubescent in lower part, more or less glabrous but with sessile glands in upper part; with 10–20 distinct internodes, the uppermost internode 2.0–4.0 cm long and equal to the next upper internode. Cauline leaves oblanceolate 10.0–30.0 × 1.0–2.0 mm, pubescent. Calyx 12.0–13.0 mm long, campanulate at anthesis and clavate in fruit, pubescent, scabrous; teeth unequal; shorter ones 1.5–2.0 mm, lanceolate, acuminate; longer ones 2.0–2.5 mm, lanceolate, acuminate; marginal hairs long (longer than 0.5 mm), dense. Inflorescence non-divaricate, branch axile (much) less than 90°. Petal claws 6.0–7.0 mm long, ciliate; limbs 4.0–5.0 mm long, bifid, upper-surface white or pink, lobes oblong, petal limbs cleft to middle or more; coronal scales ovate, apex dentate or erose. Anthophore 5.0–6.0 mm long, densely puberulent. Anthers included; filaments 6.0–7.0 mm long, glabrous or pubescent. Styles included. First pedicel 0.5–1.0 cm in flower, and 1.0 cm in fruit, erect, glabrous, apex antrorse. Capsule 7.0 mm long, oblong, fragile, opaque. Seeds 0.7–0.9 mm wide, 0.7 mm high, testa smooth.

##### Distribution.

Cyprus (Famagusta) (Fig. 7).

##### Notes.

Distinguished by its rather “leafy” appearance (even more than subsp. microsperma), due to the many short internodes (of about half to the same length as the subtending pair of leaves), the short pedicels and the short calyx teeth in comparison with the calyx tube length. Restricted to the area around Salamis and Famagusta, on the north coast of Cyprus. This subspecies is very similar to S. microsperma subsp. maritima (Boiss.) Eggens, F.Jafari & Oxelman, comb. & stat. nov. Despite the existence of morphological overlaps, S. microsperma subsp. cypria is taller and has shorter calyx.

This subspecies is nested within a clade including S. microsperma subsp. modesta (Boiss. & C.I. Blanche) Eggens, F.Jafari & Oxelman, comb. & stat. nov., *S. exsudans*, *S. chaetodonta* ID. 181 and *S. georgievskyi* ID. 42 in *RPB2* tree (PP = 0.80, Fig. 5). This subspecies is closely related to S. microsperma subsp. maritima in the chloroplast phylogeny (PP = 0.93, Fig. 4), however, the ITS phylogeny does not have enough resolution to show the closest relative of this subspecies. All subspecies of *S. microsperma* except S. microsperma subsp. cypria share a 6 bp insertion in *rps16*. The absence of this insertion, subtle morphological differences, and geographical distinction lead us to treat it as a subspecies.

#### 8c. Silene microsperma subsp. maritima

Taxon classificationPlantaeCaryophyllalesCaryophyllaceae

(Boiss.) Eggens, F.Jafari & Oxelman, comb. et stat. nov.

09DB3339-580E-5F69-AE2B-97617C8AEB94

urn:lsid:ipni.org:names:77211378-1

≡ Silene kotschyi Boiss. var. maritima Boiss., Flora Orientalis, 1: 1867. Type: [Turkey], in arenosis maritimis Ciliciae ad Mersina, 2 June 1855, *B. Balansa 801* (lectotypes, designated here: G-BOIS! [G00544628]; isolectotypes: G! [G00378630, G00378631], K! [K000728449], JE! [JE00016142, JE00016143], L [L.1713650], WAG! [WAG0004032])

##### Description.

5.0–20.0 cm tall, spreading. Stem pubescent in lower part, scabrous, pubescent with sessile glands in upper part; with 5–8 distinct internodes, the uppermost internode (0.5–)1.0–3.0 cm long and obviously longer than the next upper internode. Basal leaves oblanceolate 10.0–30.0× 1.0–3.0 mm, pubescent. Cauline leaves oblanceolate 10.0–30.0 × 1.0–3.0 mm, pubescent. Calyx 13.0–15.0 mm long, campanulate at anthesis and clavate in fruit, pubescent; teeth unequal; shorter ones 2.0–3.0 mm, lanceolate, acuminate; longer ones 2.0–4.0 mm, lanceolate, acuminate marginal hairs long (longer than 0.5 mm), dense. Inflorescence non-divaricate, branch axile (much) less than 90°. Petal claws 6.0–7.5 mm long, ciliate; limbs 5.0–6.5 mm long, bifid, upper-surface white, lobes oblong, petal limbs cleft to middle or more, lower-surface white; coronal scales 0.9–1.5 mm long, ovate, apex laciniate or dentate. Anthophore 5.0–6.0 mm long, tomentose or puberulent. Anthers exserted; filaments 6.0–9.0 mm long, sparsely pubescent . Styles slightly exserted. First pedicel 1.0–2.0 cm early flower, 1.0–2.0 cm in fruit, erect, glabrous, apex antrorse. Capsule 6.0–8.0 mm long, oblong, fragile, opaque. Seeds 0.7–0.9 mm wide, 0.4–0.7 mm high, testa smooth.

##### Distribution and habitat.

Mediterranean coasts of the Içel, Adana, and Hatay provinces (Turkey) and N Syria (Fig. 7). Growing on seashores.

##### Notes.

This taxon is readily recognized by its small size, oblanceolate leaves, and relatively long calyx. It is also characteristically tomentose. The exposed habitat (seashores) results in the calyx primary veins often to be reddish. Even though it resembles *S. exsudans* in size, habitat, leaf shape and indumentum, it is readily distinguished from this taxon by its longer (13–15 mm) calyx with longer lanceolate teeth (see also notes about *S. exsudans*). The two taxa are allopatric.

The ITS and *rps16* sequences of this subspecies are included in phylogenetic analyses, where this taxon is unresolved among others subspecies in the species and ITS trees except for the *rps16* phylogeny.

#### 8d. Silene microsperma subsp. modesta

Taxon classificationPlantaeCaryophyllalesCaryophyllaceae

(Boiss. & C.I.Blanche) Eggens, F.Jafari & Oxelman, comb. et stat. nov.

57EB0B02-1E34-56D5-B754-9E028A7C739F

urn:lsid:ipni.org:names:77211379-1

##### Description.

20.0–50.0 cm tall, erect or sometimes spreading. Stem scabrous, pubescent in lower part, scabrous, glabrous with sessile glands in upper part; with 4–10 distinct internodes, the uppermost internode 3.0–6.0 cm long and obviously longer than the next upper internode. Cauline leaves oblanceolate 10.0–40.0 × 1.0–4.0 mm, pubescent. Calyx 13.0–15.0 mm long, campanulate at anthesis and clavate in fruit, pubescent, scabrous; teeth unequal; shorter ones 2.0–3.0 mm, ovate, acuminate; longer ones 2.0–4.0 mm, lanceolate, acuminate; marginal hairs long (longer than 0.5 mm), dense. Inflorescence non-divaricate, branch axile (much) less than 90°. Petal claws 8.0–9.0 mm long, ciliate; limbs 3.0 mm long, bifid, white to pink, lobes oblong, petal limbs cleft to middle or more; coronal scales ca 1.0 mm long, ovate, apex entire or slightly erose. Anthophore 2.5–3.5 mm long, densely puberulent. Anthers included; filaments 6.0–9.0 mm long, glabrous or sparsely pubescent. Styles included. First pedicel 1.0–3.0 cm in flower, 2.0–4.0 cm in fruit, erect, glabrous, apex antrorse. Capsule 9.0–11.0 mm long, oblong or ellipsoid, robust. Seeds 0.6–0.8 mm wide, 0.6–0.7 mm high, testa smooth.

##### Distribution.

Palestine, Lebanon (Fig. 7).

##### Notes.

Distinguished by the short anthophore and long capsule that is unusually thick-walled and robust. This taxon has all the characteristics of a self-pollinating *Silene*, e.g. short anthophore, large capsule, small petal limbs, and anthers and styles included in the corolla mouth (Aydin et al. 2014b). This taxon used to be considered as closely related to *S. chaetodonta*, but the molecular phylogenies (Figs 1, 3, 5) show that “*S. modesta*” belongs in the *S. microsperma*-group. In order to emphasize this information, we have therefore decided to treat this taxon as a subspecies of *S. microsperma* rather than recognizing it as a species.

#### 9. Silene exsudans

Taxon classificationPlantaeCaryophyllalesCaryophyllaceae

Boiss. & Heldr., Diagn. Pl. Orient. 8: 76. 1849.

EF4AC7C8-6BCB-5F0B-A147-D956659953B9

##### Type.

[Turkey, Antalya] in arenosis maritimis portûs Tchinova Lyciae, [12.5.1845], *T.H.H. v. Heldreich s.n.* (lectotype, designated here: G-BOIS! [G00544614]; isolectotypes: G! [G00226916], BM! [BM000990900], E! [E00286972], LE! [LE01051364], WAG! [WAG0191880]).

##### Description.

5.0–20.0 cm tall, spreading. Stem pubescent in lower part, scabrous, pubescent with sessile glands in upper part; with 4–7 distinct internodes, the uppermost internode obviously longer than the next upper internode. Basal leaves oblanceolate or spathulate, pubescent. Cauline leaves oblanceolate 10.0–25.0 × 1.0–5.0 mm, pubescent, scabrous. Calyx 7.5–8.5 mm long, campanulate at anthesis and clavate in fruit, pubescent, scabrous; teeth unequal; shorter ones 2.0–3.0 mm, deltoid, acuminate; longer ones 2.0–4.0 mm, deltoid, mucronate; marginal hairs long (longer than 0.5 mm). Inflorescence non-divaricate, branch axile (much) less than 90°. Petal claws 5.0–6.0 mm long, ciliate; limbs 3.0–4.5 mm long, bifid, upper-surface pink, lobes ovate, petal limbs cleft to less than middle, lower-surface pink; coronal scales ca 0.5 mm long, ovate, apex dentate or erose. Anthophore 3.0–5.0 mm long, densely puberulent. Anthers included; filaments 5.0–6.0 mm long, glabrous or pubescent. Styles exserted. First pedicel 1.0–2.0 cm in flower, 1.0–3.0 cm in fruit,erect, glabrous, apex antrorse. Capsule 5.0–7.0 mm, ellipsoid, fragile, opaque. Seeds 0.7–0.8 mm wide, 0.8–1.0 mm high, testa smooth.

##### Distribution and habitat.

S Mediterranean, Turkey (Lycia) (Fig. 7). On sandy beaches near the sea.

##### Notes.

Readily distinguished by its short calyx and short, deltoid (or broadly ovate) calyx teeth from S. microsperma subsp. maritima (see also notes about that taxon), its oblanceolate leaves, ascending habit and short size of the plant. Coode and Cullen (1967) considered “*S. exsudans*” as a synonym of S. kotschyi var. maritima. Our phylogenies (Figs 1, 3, 5) verify it as belonging to the *S. microsperma*-group but as a distinct species.

We generated two ITS sequences for *S. exsudans*, which form a strongly supported clade (PP = 1.00 MPB = 90% MLB = 95%, Fig. 3) in the phylogeny. This species is nested within the unresolved *S. microsperma* clade in the ITS tree and the *RPB2* phylogeny (PP = 0.98 MPB = 86% MLB = 97%, Fig. 3, PP = 0.96 MPB = 83%, Fig. 5). The significant morphological differences lead us to treat *S. exsudans* as a distinct species instead of merging it as subspecies of *S. microsperma*.

## Conclusion

According to the current chloroplast and nuclear phylogenies, S. sect. Arenosae is a monophyletic group, and distinct from other lineages of S. sect. Rigidulae s.l. Although our ITS phylogeny does not provide sufficient resolution for the monophyly and closest relatives of S. sect. Arenosae, the ITS phylogeny based on a comprehensive sampling from the species-rich genus Silene supports the monophyly of the section. Our species tree recovers one lineage (lineage 4 in Fig. 1 which is called S. sect. Muscipula) of S. sect. Rigidulae s.l. centered in N Africa and the W Mediterranean as the closest relative of S. sect. Arenosae.

Despite the affinity between *S. chaetodonta* and one accession of *S. georgievskyi* based on the similarity matrix and phylogenies, some morphological differences lead us to retain these taxa as distinct species. The close relationship of *S. georgievskyi* ID. 42 to the clade of *S. microsperma* rather than *S. chaetodonta* and another accession of *S. georgievskyi* in the *rps16* and *RPB2* phylogenies suggests a possible hybrid origin of *S. georgievskyi*.

We propose two new combinations and status (S. microsperma subsp. maritima and S. microsperma subsp. modesta) and one new name (S. microsperma subsp. cypria).

## Supplementary Material

XML Treatment for Silene section Arenosae Eggens, F.Jafari & Oxelman, sect. nov.

XML Treatment for Silene arenosa

XML Treatment for Silene leyseroides

XML Treatment for Silene linearis

XML Treatment for S. austroiranica

XML Treatment for S. georgievskyi

XML Treatment for Silene chaetodonta

XML Treatment for Silene striata

XML Treatment for Silene microsperma

XML Treatment for Silene microsperma subsp. microsperma

XML Treatment for Silene microsperma subsp. cypria

XML Treatment for Silene microsperma subsp. maritima

XML Treatment for Silene microsperma subsp. modesta

XML Treatment for Silene exsudans

## Supplementary materials

Supplementary material 1 Material used for phylogenetic analyses Data type Table including vouchers https://binary.pensoft.net/file/447292This dataset is made available under the Open Database License (http://opendatacommons.org/licenses/odbl/1.0/). The Open Database License (ODbL) is a license agreement intended to allow users to freely share, modify, and use this Dataset while maintaining this same freedom for others, provided that the original source and author(s) are credited.Frida Eggens, Farzaneh Jafari, Mikael Thollesson, Simon Crameri, Shahin Zarre, Bengt Oxelman
