# Supplementary material for: Phylogeny and species delimitation in Silene sect. Arenosae (Caryophyllaceae): a new section
Source: PhytoKeys. 2020 Sep 4;159:1–34. doi: 10.3897/phytokeys.159.51500 (PMC7486315; doi:10.3897/phytokeys.159.51500)
Supplement: Supplementary material 1 — Material used for phylogenetic analyses [file phytokeys-159-001-s001.rtf]

Supplementary material 1
Table. Material used for phylogenetic analyses. Excluding S. sect. Arenosae, sectional circumscriptions follow Jafari et al. in press]. Herbarium abbreviations according to Thiers (2019+). Missing accessions are indicated by —; new sequences are indicated with asterisk.
Taxon and geographic origin	Voucher	Specimen ID	GenBank Accession numbers	
			ITS	Rps16	RPB2	
Silene amoena L. [S. sect. Auriculatae s.l.]						
Canada	Argus 1038 UPS	7328	—	LC423825	MT209855*	
Silene ampullata Boiss. [S. sect. Auriculatae s.l.]						
Iraq, Fuj	Al-Dabbagh and Jasim 48874 LE	6917	KX757443	—	EF061345	
Iran	Hewer 2068 GB	6180	X86869	—	MT209833*	
Iran, Kurdestan	Ghahreman 39249 TUH	18995	LC424066	LC423994	—	
Silene antirrhina L. [S. sect. Sclerophyllae]						
	M.A. Wincent and T.G. Lammers 3137 GB	7308	KX757575	Z83193	EF061318	
Silene andryalifolia [S. sect. Siphonomorpha s.l.]						
Spain, Cadiz	DSBG 4285 GB	12229	KX757481	LC423627	MT209832*	
Silene arenosa K.Koch [S. sect. Arenosae]						
Armenia, Gorovan	Tachgabzjan, Mulkidzhanjan, Gabrielian 26051960 W	182	KX757588	LC423630	MT209861*	
Armenia	Grossheim 24.vii.1919 B	45	EF060203	EF061363	EF061323	
Silene auriculata Sm. [unplaced taxon]						
Greece, Korinthos	Baden and Franzén 795 Strid	14154	KX757292	LC423636	MT209864*	
Silene austroiranica Rech. f., Aell. & Esfand. [S. sect. Arenosae]						
Oman, Jebel Akhdar	Popov 57/79 BM	10	KX757589	LC423787	MT209845*	
Iran, Tarum	Rechinger 3261 BM	11	EF060204	EF061364	EF061324	
Silene auriculata Sm. [unplaced taxon]						
Greece, Korinthos	Baden and Franzén 795 Strid	14154	KX757292	LC423636	MT209864*	
Silene berthelotiana Webb ex Christ [S. sect. Siphonomorpha s.l.]						
Spain, Tenerife	Arnoldo Santos Guerra 01/07/2004 ORT	12235	KX757495	LC423641	MT209830*	
Silene bupleuroides L. [S. sect. Sclerocalycinae s.l.]						
Garden	Bengt Oxelman 2392 UPS	6286	—	EF061360	EF061319	
Supplementary material 1. Table (continued).
Taxon and geographic origin	Voucher	Specimen ID	GenBank Accession numbers	
			ITS	Rps16	RPB2	
Silene burchellii Otth [S. sect. Silene s.l.]						
Garden	Bengt Oxelman 2280 GB	2490	X86868	LC423976	MT209839*	
Silene cappadocica Boiss. & Heldr. [S. sect. Auriculatae s.l.]						
Iran	Wendelbo and Assadi 28000 GB	7534	KX757451	EF061383	EF061346	
Silene cariensis Boiss. [S. sect. Sclerocalycinae s.l.]						
Turkey, Mugla	Bengt Oxelman 1681 GB	1168	EF060205	EF061365	EF061325	
Silene chaetodonta Boiss. [S. sect. Arenosae]						
Iraq, Sulaimaniya	Rechinger 10060 W	7561	KX757596	LC423656	MT209843*	
Iraq, Sulaimaniya	Rechinger 10146 W	6259	KX757595	LC423781	MT209842*	
Iran, Shahrud-Bustam	Rechinger 51024 W	7642	KX757597	—	—	
Turkey	Huber-Morath 10709 E	181	—	—	MT209847*	
Silene congesta Sm. [S. sect. Siphonomorpha s.l.]						
Greece	Walter Gutermann 35300 Walter	13310	KX757514	KY045505	MT209831*	
Silene cuatrecasasii Pau & Font Quer [S. sect. Muscipula]						
Morocco, Al Hoceima	Optima Iter V 1553 RNG	7638	EF060207	EF061367	EF061327	
Silene echinosperma Boiss. & Heldr. [S. sect. Rigidulae s.str.]						
Greece, Lakonias	Bengt Oxelman 2225 GB 	2430	X86845	Z83196	EF061330	
Silene echinospermoides Huber-Mor. [S. sect. Rigidulae s.str.]						
Greece, Rhodes	Bengt Oxelman 2202 GB	2407	KX757636	EF061368	EF061328/EF061329	
Silene microsperma exsudans Boiss. & Heldr. [S. sect. Arenosae]						
Turkey, Mughla	Bengt Oxelman 1660 GB	1147	EF060209	EF061369	EF061331	

Supplementary material 1. Table (continued).
Taxon and geographic origin	Voucher	Specimen ID	GenBank Accession numbers	
			ITS	Rps16	RPB2	
Turkey, Antalya	Bengt Oxelman 1706 GB	1192	KX757598	—	—	
Silene fabaria (L.) Sm. [S. sect. Behenantha s.l.]						
Greece, Samou	Bengt Oxelman, 2211 GB	2416	X86851	LC423681	HM595240	
Silene fruticosa L. [S. sect. Siphonomorpha s.l.]						
Greece, Lakonias	Bengt Oxelman and Lars Tollsten 934 GB	1064	KX757508	LC423685	AJ634078	
Silene fuscata Link ex Brot. [S. sect. Silene s.l.]						
Algeria	Bengt Oxelman 1887 GB	1348	X86837	LC423686	MT209838*	
Silene georgievskyi Lazkov [S. sect. Arenosae]						
Iraq, Western Desert	Rechinger 9828 B, G	41	KX757608	LC423780	—	
Syria (?), Antilibanon	Rechinger 13136 G	42	KX757609	LC423688	MT209846*	
Silene hawaiiensis Sherff [S. sect. Sclerophyllae]						
USA, Hawaiian Islands	Degener, Greenwell and Murashige 19828 GB	12555	EF060218	EF061378	EF061340	
Silene imbricata Desf. [S. sect. Silene s.l.]						
Algeria	Bengt Oxelman 1881 GB	1344	KX757266	LC423700	MT209840*	
Silene inaperta L. [S. sect. Muscipula]						
Spain, Malaga	Bengt Oxelman 1724 GB	1288	EF060210	EF061370	EF061332	
Portugal	Silva 1688 LD	5360	KX757584	LC423698	MT209860*	
Silene kuhistanica Ovcz. [S. sect. Auriculatae s.l.]						
Afghanistan, Balkh	Grey-Wilson and Hewer 846 GB	7367	KX757441	—	MT209837*	
Silene leyseroides Boiss. [S. sect. Arenosae]						
Iran, Fars	Kramer 14635 BSB	160	EF060211	EF061371	EF061333	
Iraq, Faluja Desert	Wheeler Haines 164 E	7318	KX757613	LC423789	MT209862*	

Supplementary material 1. Table (continued).
Taxon and geographic origin	Voucher	Specimen ID	GenBank Accession numbers	
			ITS	Rps16	RPB2	
Afghanistan, Qataghan	Rechinger 16510 C	6256	KX757612	LC423788	MT209853*	
Kuwait, Al-Subiyah	Boulos 15500 S	12072	KX757614	LC423710	MT209863*	
Silene linearifolia Otth [S. sect. Siphonomorpha s.l.]						
Georgia	Hörandl et al C88-31 WU	12881	KX757580	LC423711	MT209834*	
Silene linearis Decne [S. sect. Arenosae]						
Jordan, Dead Sea	Davis 3754 E	50	KX757591	LC423779	MT209854*	
Jordan, Dead Sea	M. Bierkamp and P. Zinth 177 BSB	155	EF060212	EF061372	EF061334	
Egypt, Read Sea coast	Schweinfurth 779 BM	49	KX757593	—	—	
Silene martyi Emberger & Maire [S. sect. Muscipula]						
Morocco, Tetouan	F.Jacquemoud and D.Jeanmonod no.MAR1168 G	52	EF060213	EF061373	EF061335	
Silene mentagensis Coss. [S. sect. Portenses]						
Morocco	Jahandiez 329 LD	12071	EF060236	EF061396	EF061358	
Silene microsperma Fenzl subsp. cypria Eggens & Oxelman [S. sect. Arenosae]						
Cyprus, Salamis	Julin 26.VI.1971 UPS	7535	KX757603	LC423786	MT209848*	
Silene microsperma Fenzl subsp. maritima (Boiss.) Eggens & Oxelman [S. sect. Arenosae]						
Turkey, Içel	Nydegger 40480 B	14	KX757605	LC423782	—	
Silene microsperma Fenzl subsp. microsperma [S. sect. Arenosae]						
Turkey, Adana	Aberdeen Univ. Amanus Exp. D3356 E	2992	KX757599	LC423783	MT209850*	
Turkey, Malatya	Stainton and Henderson 5492 E	7648	KX757600	LC423785	MT209852*	
Turkey, Malatya	Sorger 73-30-6 W	7647	KX757602	LC423784	MT209851*	

Supplementary material 1. Table (continued).
Taxon and geographic origin	Voucher	Specimen ID	GenBank Accession numbers	
			ITS	Rps16	RPB2	
Turkey, Malatya	Davis, Dodds & Cetik 20402 E	3371	KX757606	—	—	
Silene microsperma Fenzl subsp. modesta (Boiss. & Blanche) Eggens & Oxelman [S. sect. Arenosae]						
Palestine, Jaffa	Samuelsson 777 S	12055	KX757604	LC423719	—	
Palestine, Jaffa	J. Bornmüller 193 G	5640	KX757601	—	MT209849*	
Silene moorcroftiana Wall. ex Bentham [S. sect. Auriculatae s.l.]						
Tajikistan, Gorno-Badakhshan	B. Dickoré 17783 MSB	11380	EF060229	EF061388	EF061351	
Silene muscipula L. [S. sect. Muscipula]						
Morocco, Djebel Azrou Achkar	Bengt Oxelman 1780 GB	1271	EF060201	Z83197	EF061320	
	Chevalier 548 WU	12865	—	—	MT209841*	
Silene nana Kar. & Kir. [S. sect. Saponarioides]						
Turkmenistan	Kereverzova and Mekeda 1976.V.5 LECB	6787	KX757312	EF061377	EF061339	
Silene noctiflora L. [S. sect. Elisanthe]						
Garden	H.C. Prentice N2-2/14327 BOX	14327	FN821141	FN821309	HM595245	
Silene nocturna L. [S. sect. Silene s.l.]						
Greece, Fokidos	Bengt Oxelman and Lars Tollsten 654 GB	1088	X86841	Z83192	AJ634083	
Silene nutans L. [S. sect. Siphonomorpha s.l.]						
Garden	Magnus Popp 1045 UPS	7610	KX757501	EF061361	EF061321	
Silene odontopetala Fenzl [S. sect. Odontopetalae]						
Turkey	Görk et al. 23817 Strid	14169	KX757294	LC423726	MT209865*	
Silene papillosa Boiss. [S. sect. Auriculatae s.l.]						
Turkey, Mughla	Bengt Oxelman 1678 UPS	1165	KX757465	—	MT209856*	
Silene pendula L. [S. sect. Behenantha s.l.]						
Supplementary material 1. Table (continued).
Taxon and geographic origin	Voucher	Specimen ID	GenBank Accession numbers	
			ITS	Rps16	RPB2	
Garden	Anja Rautenberg 289 UPS	12654	FN821142	FN821310	HM595246	
Silene pinetorum subsp. sphaciotica Oxelman and Greuter [S. sect. Rigidulae s.str.]						
Greece, Crete	Bengt Oxelman et al. 2183 GB	2383	KX757634	LC423971	MT209868*	
Silene portensis L. [S. sect. Portenses]						
Garden	Anja Rautenberg 39 UPS	6283	—	—	MT209859*	
Spain	Sanchez Garcia 1974.VI.17 C	7003	EF060237	EF061397	EF061359	
Silene portensis L. subsp. maura Emb. & Maire [S. sect. Portenses]						
Morocco	Marim, A. Mêtro, Ch. Sauvage 951 S	11775	—	LC423970	MT209857*	
Morocco	Podlech 46825 G	136	KX757645	LC423969	MT209858*	
Silene pseudoatocion Desf. [S. sect. Silene s.l.]						
Garden	Erixon 71 UPS	7821	—	EU314656	FJ376918	
Silene reinwardtii Roth [S. sect. Rigidulae s.l.]						
Turkey, Yaylakonak	Bengt Oxelman 2391 UPS	2602	EF060215	EF061375	EF061337	
Turkey, NW Antalya	Kehl 28.VI.1979 B	5468	KX757639	LC423735	KX757475	
Jordan	T. Engel, Frey and H. Kürschner 90-223 BSB	110	KX757637	LC423819	MT209835*	
Silene reticulata Desf. [S. sect. Muscipula]						
Algeria, Blida	Davis 53457 BM	3357	EF060216	EF061376	EF061338	
Silene rhynchocarpa Boiss. [S. sect. Auriculatae s.l.]						
Turkey	Hörandl et al 4767 WU	12888	—	LC423827	MT209836*	
Silene schafta S.G.Gmel. ex Hohen. [S. sect. Auriculatae s.l.]						
Iran, Mazandaran	A. Gholipour 4706 SPNH 	19029	LC424078	LC424002	—	
Supplementary material 1. Table (continued).
Taxon and geographic origin	Voucher	Specimen ID	GenBank Accession numbers	
			ITS	Rps16	RPB2	
Garden	Magnus Popp 1053 UPS	7615	AJ831792	—	AJ634088	
S. striata Ehrenb. ex Rohrb. [S. sect. Arenosae]						
Syria, Damascus	Samuelsson 1522 S	12068	KX757610	LC423745	MT209844*	
Silene turkestanica Regel [S. sect. Elisanthe]						
Kyrgyzstan	G.A. Lazkov 12.VII.2001 FRU	12131	FN821146	FN821314	MT209866*	
Silene ungeri Fenzl [S. sect. Rigidulae s.str.]						
Greece, Ioanninon	Bengt Oxelman and Lars Tollsten 1431 GB	499	EF060202	EF061362	EF061322	
Silene uralensis (Rupr.) Bocquet [S. sect. Physolychnis s.l.]						
Garden	(Rupr.) Bocquet SUP02-38-8 UPS	7023	KX757342	AJ831768	AJ634206	
Silene vittata Stapf [S. sect. Sclerocalycinae s.l.]						
Turkey, Antalya	Bengt Oxelman 2390 UPS	2601	KX757643	LC423749	MT209867*	
Silene viscosa (L.) Pers [S. sect. Physolychnis s.l.]						
Garden	Anja Rautenberg 104 UPS	7705	FN821148	FN821316	HM595251	
Silene vulgaris subsp. angustifolia (Mill.) O.Bolòs & Vigo [S. sect. Behenantha s.l.]						
Spain, Malaga	Mats Thulin 5717 UPS	6459	FN821149	FN821317	HM595252	
